# Phototunable Chiral-Selective Nanoporous Material

**DOI:** 10.1021/acsami.5c14743

**Published:** 2025-10-21

**Authors:** Iván Marín, Pilar López Ram de Viu, Pilar Romero, Danqing Liu, Dirk J. Broer, Joaquín Barberá, José Luis Serrano

**Affiliations:** † Instituto de Nanociencia y Materiales de Aragón (INMA), CSIC-Universidad de Zaragoza, 50009 Zaragoza, Spain; ‡ Departamento de Química Orgánica, Facultad de Ciencias, 16765 Universidad de Zaragoza, 50009 Zaragoza, Spain; § Instituto de Síntesis Química y Catálisis Homogénea (ISQCH), CSIC-Universidad de Zaragoza, 50009 Zaragoza, Spain; ∥ Department of Chemical Engineering and Chemistry Eindhoven, University of Technology, Groene Loper 3, Eindhoven 5612AE, The Netherlands

**Keywords:** chiral nanoporous materials, enantioselective absorption, phototunable chirality, H-bonded supramolecular complexes, hexagonal columnar phases

## Abstract

Herein, we present the development of tunable chiral nanoporous materials capable of enantioselectively adsorbing racemic mixtures. The materials are based on hydrogen-bonded supramolecular structures formed from a melamine derivative as the central core and three peripheral dendrons functionalized with coumarin and azobenzene moieties that are organized in hexagonal columnar phases. Upon irradiation with circularly polarized light (CPL), the azobenzene units induce controlled chirality in the material, which is stabilized by photodimerization of the coumarin moieties, obtaining mechanically stable films. The elimination of the melamine template results in nanoporous materials that retain their homeotropic hexagonal columnar alignment. These materials show selective adsorption of one particular enantiomer of racemic mixtures of (hexan-2-yl)-4-nitroaniline. Pore chirality can be reversed by changing the handedness of the CPL, allowing nanoporous materials to adsorb predominantly one or the other enantiomer. These findings offer a versatile strategy for enantioselective separation with potential applications in pharmaceuticals and materials science.

## Graphical abstract

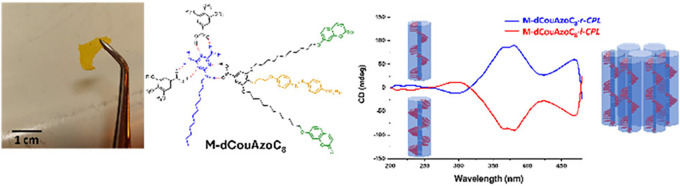

## Introduction

Chirality in materials is crucial in fields as different as life sciences^1,2^ (i.e., biology, pharmacy) and materials science;^3–6^ however, even today, the generation of enantiomer-pure compounds remains a challenge.^7–9^ Among the strategies used in the purification of molecular compounds, nanoporous materials based on liquid crystal (LC) arrangements, both calamitic and columnar, have emerged as powerful tools for the separation of molecules by size, charge, or dispersive affinity. In many cases, the characteristic LC molecular organization is stabilized by photopolymerization using a wide variety of tethered reactive groups, such as methacrylates, acrylates, dienes, epoxides, and oxetanes.^10–22^ Our group published the first example of macroscopic fixation of a columnar structure through photodimerization of coumarins, obtaining polymeric nanomembranes that adsorb–reject molecules by size and/or charge.^23^ Coumarins photopolymerize, without the need for a catalyst, when they are irradiated with 325 nm light.^24–27^ Besides this, in their liquid crystal phase, these disk-like coumarin-containing molecules adopt often a homeotropic alignment, with the column axes perpendicular to the film surface,^28^ which could potentially favor the pore alignment necessary for the molecular separation in a preferential direction.

To use this strategy for the separation of chiral compounds, we need to introduce chirality into the nanoporous membranes. This can be achieved through chiral groups producing permanent chirality in the material.^29–32^ On the other hand, another interesting approach, without chiral groups, is based on the introduction of photosensitive reactive groups capable of generating chirality when irradiated with circularly polarized light.^33,34^ Specifically, irradiation of integrated azobenzene groups with left- or right-handed circularly polarized light generates helical structures with the desired handedness.^35–40^ This method also has been used in the preparation of MOF nanoporous materials with chiral selectivity.^41^

In order to improve further the versatility of this approach, in this work, we describe the preparation of a nanoporous material with photoswitchable chirality for the selective adsorption of stereoisomers. The strategy we used is based on the preparation of a mechanically stable nanoporous structure from an azo-containing columnar liquid crystal. The pores organize into a unique and controlled helical morphology when they are irradiated with circularly polarized light. Thereto, we design a disk-shaped supramolecular structure formed from a melamine derivative (M) acting as the central core, which self-assembles, through double hydrogen bonds, with three dendrons that carry carboxylic groups in their apical position (See Figure 1a). These dendrons have two lateral branches functionalized with coumarin groups in their terminal positions and a promesogenic azo group in the central branch (dCouAzoC_8_). In this way, this dendronic structure combines an azo group capable of inducing chirality when it is irradiated with circularly polarized light and photodimerizable coumarin residues that allow stabilizing the macroscopic structure of the material. A crucial aspect in the design of this dendron is the introduction of a flexible undecamethylene (CH_2_)_11_ chain in the branches of the coumarins that facilitates the accessibility of these groups in the photo-cross-linking process, and the oxyethylene group as a spacer in the branch of the azo group, that favors the mobility of this group in the switching process.

**Figure 1:**
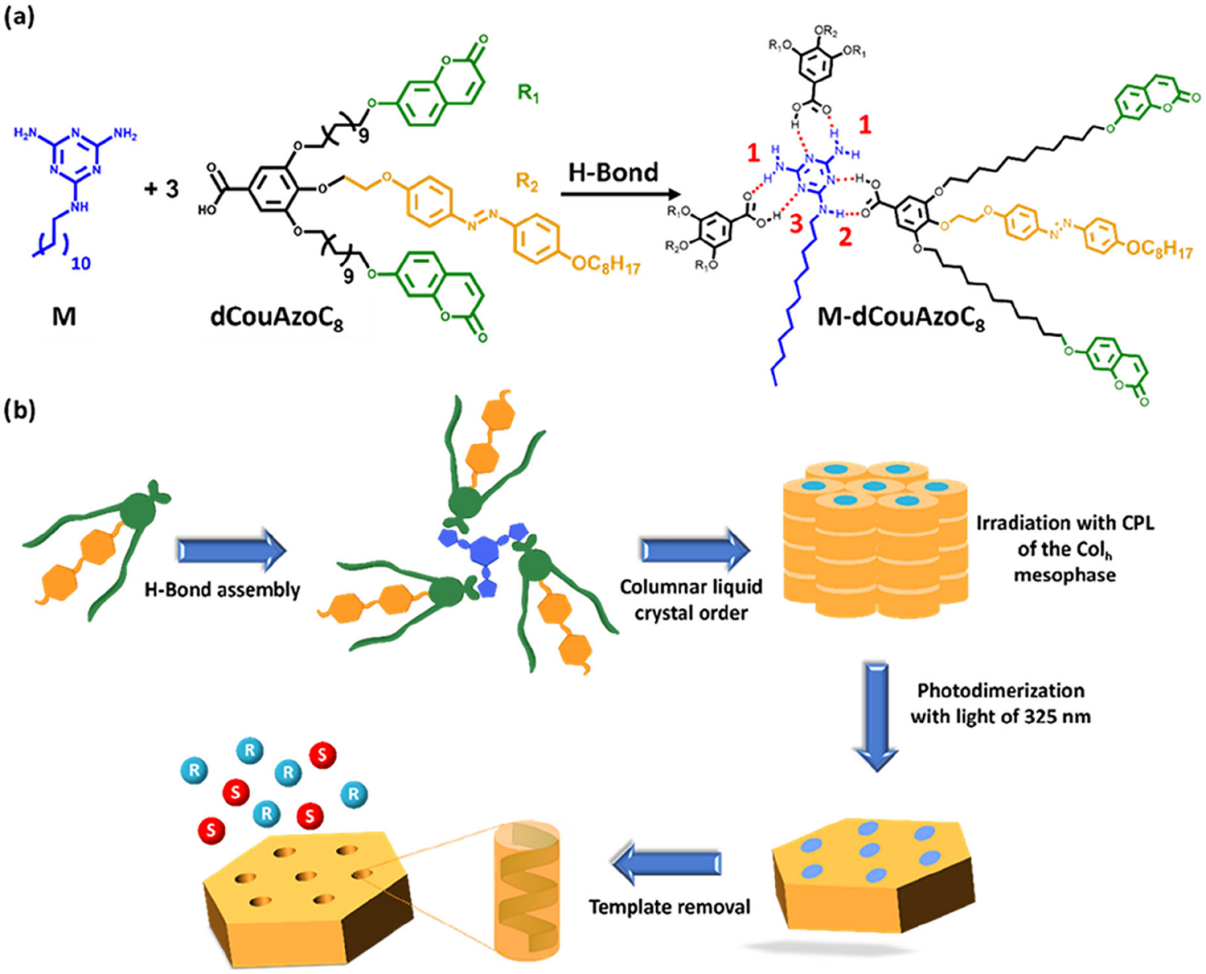
(a) Schematic representation of the melamine template (M), the lateral dendron **dCouAzoC**_**8**_ and the supramolecular complex **M-dCouAzoC**_**8**_. (b) Schematic illustration of the preparation of chiral nanoporous materials.

The strategy for the preparation of these chiral nanoporous materials is shown in Figure 1b. First, the formation of the supramolecular complex is carried out between the melamine template and dendronic moieties. Second, chirality is generated in the complex by irradiation with circularly polarized light. The third step is the stabilization of this structure by photo-cross-linking of the coumarin units, obtaining a plastic material. The fourth step is melamine template removal by acid treatment, obtaining the chiral nanoporous material. Finally, the chiral adsorption/rejection analysis and the study of the enantioselective separation of a racemic mixture are made in the obtained chiral nanomaterial and its reverse.

## Experimental Section

### Materials and Methods

#### Materials

All reagents were purchased from Aldrich and used without further purification. Anhydrous CH_2_Cl_2_ and THF were purchased from Scharlab and dried using a solvent purification system.

#### Techniques

^1^H NMR and ^13^C NMR spectra were acquired on a Bruker AV400 spectrometer. The experiments were performed at room temperature in different deuterated solvents (CDCl_3_, CD_2_Cl_2_, or DMSO-d6). Chemical shifts are given in ppm relative to TMS, and the solvent residual peak was used as the internal standard.

Infrared spectra were recorded on a Bruker Vertex 70 fourier transform infrared spectroscopy (FTIR) spectrometer. The samples were prepared on KBr pellets with a concentration of the product of 1–2% (w/w).

MALDI-TOF mass spectrometry was performed on an Autoflex Bruker mass spectrometer with a dithranol matrix. Positive and negative ion electrospray ionization high resolution (ESI HRMS) was performed on a Bruker Q-TOF-MS instrument in a positive or negative ESI mode.

Elemental Analysis was performed using a PerkinElmer 2400 microanalyzer.

Mesogenic behavior was investigated by polarized light optical microscopy (POM) using an BH-2 polarizing microscope fitted with a Linkam THMS600 hot stage and a Linkam TMS94 controller.

Thermogravimetric analysis (TGA) was performed using a Q5000IR from TA Instruments at a heating rate of 10 °C min-1 under a nitrogen atmosphere.

Thermal transitions were determined by differential scanning calorimetry (DSC) using a DSC Q2000 from TA Instruments with powdered samples (2–5 mg) sealed in aluminum pans. The apparatus was previously calibrated with indium (156.6 °C and 28.44 J/g). Glass transition temperatures (Tg) were determined at the half height of the baseline jump, and first-order transition temperatures were read at the maximum of the corresponding peak.

Ultraviolet–visible (UV–Vis) adsorption spectra were recorded on an ATI-Unicam UV4-200 spectrophotometer.

Circular dichroism spectra were recorded on a Jasco J-810 spectropolarimeter. CD spectra were recorded at different rotation angles around the light beam showing the same trace; in the graphs, the average of the six rotations were showed.

X-ray diffraction measurements were carried out using an XRD-PAN analytical Empyrean diffractometer equipped with a platform Scatter X78. Photographic patterns were recorded with a Pinhole camera (Anton Paar) operating with a point-focused Ni-filtered Cu–Kα beam. Samples were contained in Lindemann glass capillaries (0.9 or 0.7 mm diameter), and when necessary, a variable-temperature attachment was used to heat the sample. The patterns were collected on a flat photographic film perpendicular to the X-ray beam. X-ray diffraction in films (XRD) images were recorded on a Ganesha lab instrument equipped with a Genix-Cu ultralow divergence source producing X-ray photons with a wavelength of 1.54 Å and a flux of 1 × 108 photons/second. Diffraction patterns were collected on a Pilatus Nanoporous polymer | 59 300 K detector with a reversed-biased silicon diode array sensor. The detector contains 487 × 619 pixels of 172 × 172 μm^2^ and consists of three modules with an intermodule gap of 17 pixels in between, resulting in two dark bands on the image. Grazing incidence X-ray scattering (GIXS) measurements were performed on a sample to detector distance of 1080 (WAXS) or 1530 mm (SAXS). Temperature-dependent measurements were executed with a Linkam HFSX350 heating stage and a cool unit. Azimuthal integration of the obtained diffraction patterns was performed by utilizing SAXSGUI software. The beam center and the q-range were calibrated by utilizing silver behenate (0.107 Å-1; 58.43 Å).

Photo-cross-linking of coumarin units (photodimerization) was carried out by exposing the aligned LC films of 10 μm thickness to 325 nm LED light (ThorsLab) for 180 min with a UV power of 8 mW/cm^2^. CPL irradiation films were irradiated for 1 min with the corresponding CPL from the 488 nm line of an Ar+ laser, power 20 mW/cm^2^ with a polarizer of the appropriate wavelength.

X-ray photoelectron spectroscopy (XPS) spectra were recorded on a Kratos AXIS ultra DLD spectrometer equipped with an Al Kα X-ray monochromatic source (1486.6 eV) and using 20 eV as the pass energy. Binding energies were calibrated according to the C 1s peak at 284.6 eV.

Chiral HPLC: Analysis were performed on an HPLC Waters 600 system that includes a Waters Delta 600 multisolvent quaternary gradient pump, a Waters 2996 photodiode detector (PDA) and a Rheodyne 7125 injector. Data were acquired and processed with Waters Empower software.

The employed column was a Chiralpak IA tris packed 3,5-dimethylphenylcarbamate cellulose immobilized on silica, size particle (5 μm) with dimensions 250 × 4.6 mm^2^ ID. Eluent: a 90/10 hexane/EtOH (ethanol) mixture with a flow of 1 mL/min. Detection: UV adsorption at 380 nm. Ten microliters were injected directly from the solution obtained after the extraction of the samples with 1 mL of AcOEt (ethyl acetate).

#### Synthesis of Precursors

The synthesis and chemical characterization of the melamine derivative N^2^-dodecyl-1,3,5-triazine-2,4,6-triamine (**M**) acting as template have been described in previous works^23,42^ and are gathered in Section 1.1 of the SI (see Scheme S1 and Figures S1,S2 in the SI). The synthesis and chemical characterization of the lateral dendrons are described in Section 1.2 of the SI. In the first step the (*E*)-1-(4-(2-bromoethoxy)­phenyl)-2-(4-(octyloxy)­phenyl)­diazene (**AzoC**_**8**_) derivative precursor is synthesized (See Section 1.2.1, Scheme S2, and Figures S3,S4 in the SI). In the second step, the precursor bearing the photodimerizable coumarin unit 7-(11-bromoundecyloxy)-2H-chromen-2-one is prepared (see Section 1.2.2, Scheme S3. and Figures S5,S6 in the SI).^28^ Finally, the **dCouAzoC**_**8**_ side dendron is prepared by a stepwise functionalization of methyl gallate. First, one molecule of **AzoC**_**8**_ is introduced at the 4-position taking advantage of the higher reactivity of that position for Williamson etherification using a weak basic medium (KHCO_3_/KI/DMF). Second, two molecules of the derivative carrying the coumarin unit are introduced at the 3- and 5-positions also by Williamson etherification using K_2_CO_3_/KI/DMF as a basic medium. Finally, saponification of methyl gallate yields the carboxylic functionalized side dendron (see Section 1.2.3, Scheme S4, and Figures S7–S9 in the SI).

## Results and Discussion

### Synthesis of the Supramolecular Complex M-dCouAzoC_8_

The preparation of the supramolecular complex was carried out by dissolving in dichloromethane (DCM) a mixture in a ratio 1:3 of the melamine derivative (**M**) (bearing a dodecyloxy alkyl chain to favor their solubility), that acts as template core,^40^ and three molecular dendrons (**dCouAzoC**_**8**_). The solvent was slowly evaporated under continuous stirring at room temperature, yielding the supramolecular complex [**M­(dCouAzoC**_**8**_)] (see Section 2.1 and Figures S10,S11 in the SI).

The formation of intermolecular hydrogen bonds in the supramolecular complex was characterized by infrared spectroscopy (FTIR) and nuclear magnetic resonance (^1^H NMR and ^13^C NMR) in a CD_2_Cl_2_ solution. The FTIR spectra of the **dCouAzoC**_**8**_ dendron and the **M­(dCouAzoC**_**8**_) supramolecular complex are shown in Figure 2a. The interaction between the carboxylic acid of the dendron and the amino group of the template produces a shift of the C=O band of the acid from 1677 cm^–1^, corresponding to the dimeric form existing in the dendron (**dCouAzoC8**), to 1693 cm^–1^ in the complex produced by the H-bond between the carbonyl group of the dendron and the NH of the melamine (see **β** bands in Figure 2a). Likewise, significant shifts occur in the peaks observed in the ^1^H NMR spectra in CD_2_Cl_2_ at 25 °C of **dCouAzoC**_**8**_, **M-dCouAzoC**_**8**_, and **melamine** (**M**) derivatives corresponding to the protons of the carboxyl and amino groups involved in the formation of H bonds in the supramolecular complex (Figure 2b). Thus, the NH (***H***_**1**_ and ***H***_**2**_) protons of melamine (see Figure 1a for the molecular structure and **2b** for proton assignation) move to lower fields in the complex; in the melamine, these protons appear as three wide peaks from 4.85 to 4.65 ppm (the two peaks at lower frequency correspond to the ***H***_**1**_ protons and the peak at higher frequency correspond to the ***H***_**2**_ proton), whereas in the complex two signals appear at 6.00 and 6.27 ppm as it is described in the literature.^42^ The broad band corresponding to the two unassociated NH protons of melamine appears around 4.20–3.70 ppm in the spectrum of the complex (Figure S10 in the SI). Also, a shift in the protons of the α-methylene protons ***H***_**3**_ (see Figure 1a for the molecule structure and **2b** for proton assignation) of the dodecyloxy group attached to the amino group from 3.29 to 3.36 ppm is observed. In the ^13^C NMR spectra in CD_2_Cl_2_ at 25 °C, a shift in the carbon signal of the carboxyl group is observed from 170.30 ppm in the dendron to 170.49 ppm in the supramolecular complex (see signal **A** in Figure S12 in Section 2.1 of the SI).

**Figure 2:**
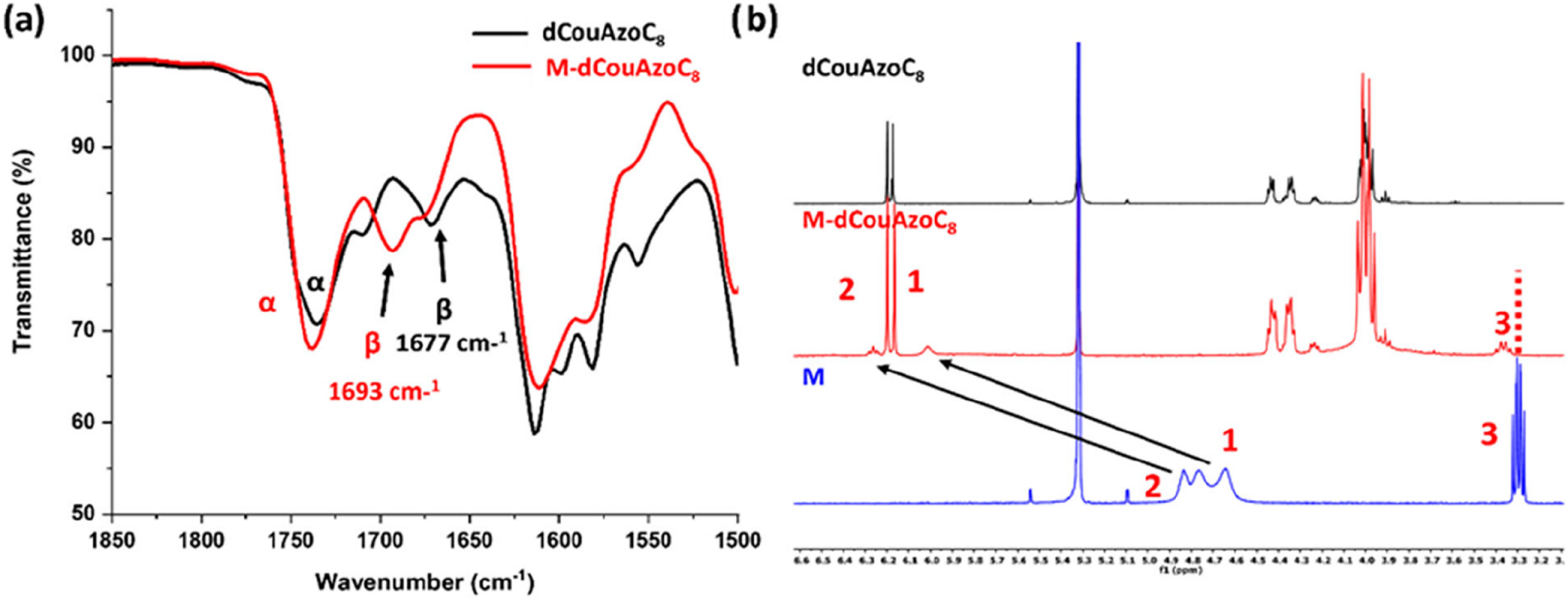
(a) FTIR comparison of **dCouAzoC**_**8**_ and **M-dCouAzoC**_**8**_. The **β** signal corresponds to the C=O of the carboxylic acid of the dendron, whereas the **α** signal corresponds to the C=O of the coumarin units see the molecular structure in Figure 1a and (b) ^1^H NMR in CD_2_Cl_2_ at 25 °C of **dCouAzoC**_**8**_, **M-dCouAzoC**_**8**_, and **melamine** (**M)** derivatives see the proton assignation in the molecular structure in Figure 1a.

The continuous variation method was applied to ^**1**^ **H NMR** experiments on complex **M-dCouAzoC**_**8**_. The shifts of **NH** and **CH**_**2**_ **-N** signals with increasing acid concentration, while keeping the melamine concentration constant, were fitted to a 1:1 stoichiometry in the CD_2_Cl_2_ solution. The binding constant of 825.8 ± 77.1 L mol-1 was calculated by nonlinear curve fitting of the chemical shifts. Data are gathered in Section 2.2 of the SI (Figures S13, S14 and Table S1).

**The thermal properties and mesogenic behavior** of the dendronic structure **dCouAzoC**_**8**_ and the supramolecular complex were studied by thermogravimetry (TGA), differential scanning calorimetry (DSC), polarization optical microscopy (POM) with a heating stage, and X-ray scattering. The most significant data are collected in Table 1.

**Table 1:** Thermal Characterization and Structural Parameters

compound	T_2%_ [°C]^a^	phase transitions^b^	d[Å]^c^	h k l^d^	structural parameter^e^,^f^
dCouAzoC_8_	320	Cr 115 I			
M-dCouAzoC_8_	245	g 31 Col_h_ 100 I^g^	43.3	1 0 0	a = 50.1 Å
			24.9	1 1 0	
			21.6	2 0 0	
			13.2	2 1 0	
			4.1	br	

a Temperature of 2% mass loss obtained by thermogravimetry.

b DSC data obtained in the cooling process at a rate of 10 °C/min, Cr: crystal, g: mesomorphic glass, Col_h_: columnar hexagonal mesophase, I: isotropic liquid.

c Observed X-ray reflections.

d Miller indices of the X-ray reflections.

e a = (2/√3)·(d_10_ + √3·d_11_ + √4·d_20_ + √7·d_21_ +...)/n_reflections_.

f V = a^2^·√3/2·c·10^–24^.

The temperature of 2% mass loss obtained by thermogravimetry is significantly higher than that of the clearing points, confirming the stability of both structures. The assignment of the mesophase was carried out using POM, MAXS (medium-angle X-ray scattering), and WAXS (wide-angle X-ray scattering) (Figure 3a–d). In POM, after subjecting the sample to mechanical stress, a granular texture compatible with a columnar mesophase is observed (Figure 3a). If the sample is allowed to cool slowly from the isotropic temperature, very large dark areas are observed, indicating a homeotropic alignment (see Figure S15 in Section 2.3 of the SI). The columnar phase is confirmed with the MAXS and WAXS diffractograms (Figure 3b–d) where two intense and fine peaks are observed at 43.3 and 24.9 Å in a 1:1/√3 spacing ratio in the low-angle region. Two other reflections are observed with a ratio of 1/√4:1/√7 with respect to the main peak ((200) and (210) planes), confirming the hexagonal columnar (Col_h_) nature of the mesophase. The large angle region contains a broad diffuse halo typical of the interactions of alkyl chains at 4.1 Å. The WAXS diffractogram also provides information on the direction of the stacking of the supramolecular complex, deduced from the presence of several discrete spots instead of circular diffractions in the low-angle region (Figure 3c), indicating a homeotropic orientation of the material (i.e., the columns are perpendicular to the glass surface). Besides this, the X-ray structural parameter measured in the columnar hexagonal phase agrees with the stoichiometry 1:3 proposed for these type of complexes (see Section 2.4 of the SI).

**Figure 3:**
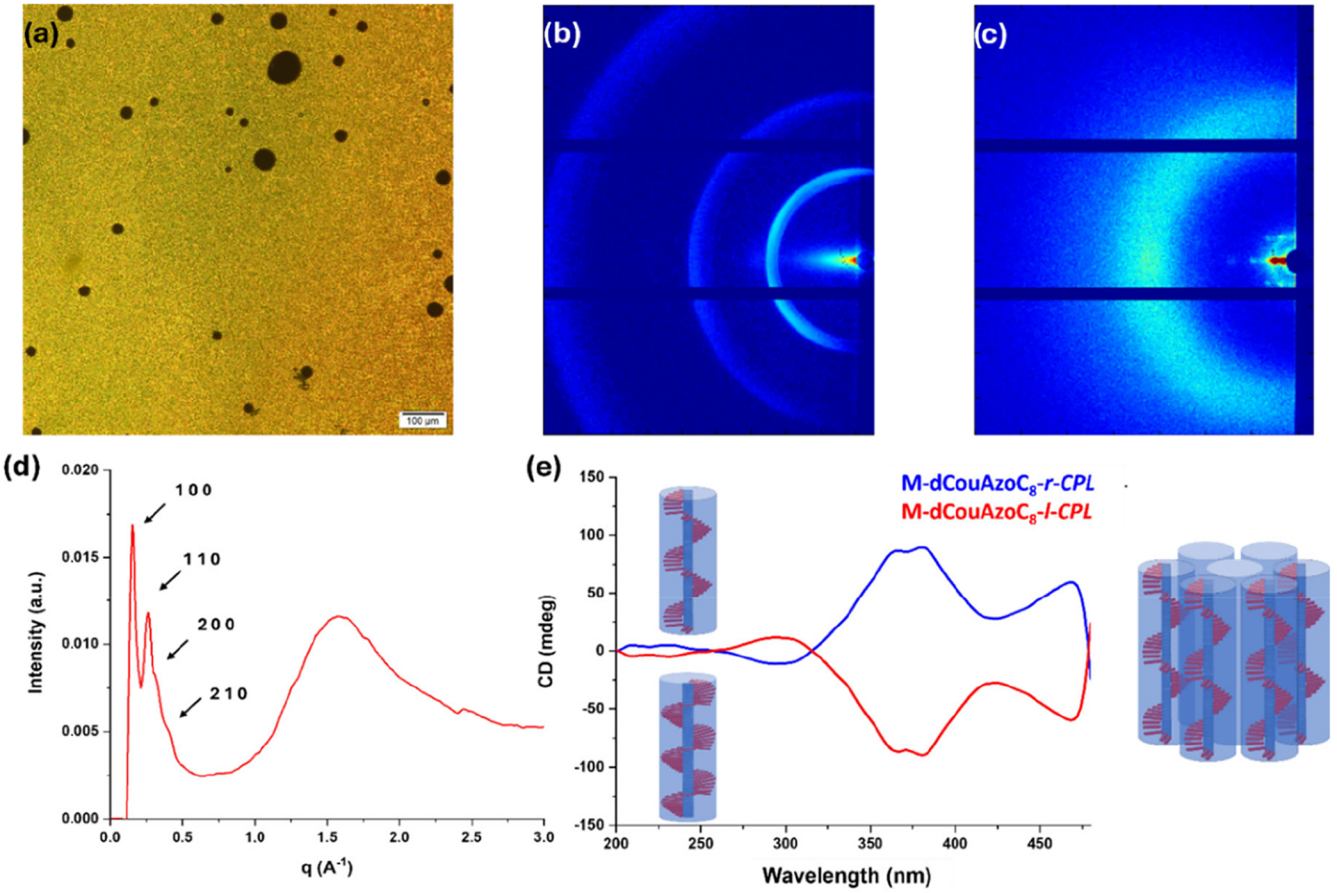
(a) POM microphotograph of **M-dCouAzoC**_**8**_ taken at 55 °C in the first cooling process, (b) 2D MAXS pattern of **M-dCouAzoC**_**8**_, (c) 2D WAXS pattern of **M-dCouAzoC**_**8**_, (d) 1D XRD pattern of **M-dCouAzoC**_**8**_, and (e) circular dichroism spectra of **M-dCouAzoC**_**8**_ after being irradiated with right circular polarized light **M-dCouAzoC**_**8**_ **-r-CPL** (blue line) and left circular polarized light **M-dCouAzoC**_**8**_ **-l-CPL** (red line).

### Preparation and Characterization of the Chiral Nanoporous Materials: M-dCouAzoC_8_-r-CPL-pol and M-dCouAzoC_8_-l-CPL-pol

The preparation of the nanoporous material involves three steps as described in Scheme S5 in Section 3.1 of the SI.

In the first step, the irradiation of **M-dCouAzoC**_**8**_ in the columnar hexagonal phase with circular polarized phase induce the helical organization in the column yielding the complexes **M-dCouAzoC**_**8**_ **-r-CPL** and **M-dCouAzoC**_**8**_ **-l-CPL** (see Section 3.2in the SI).

The second step is the preparation of the polymeric structures derived by **M-dCouAzoC**_**8**_ **-r-CPL** and **M-dCouAzoC**_**8**_ **-l-CPL** for photodimerization of the coumarin units: **M-dCouAzoC**_**8**_ **-r- CPL-pol** and **M-dCouAzoC8-l-CPL-pol** (see Section 3.3 in the SI).

Finally, the last step is the preparation of the nanoporous material by removal of the template molecules in the polymeric precursors: **M-dCouAzoC**_**8**_ **-r-CPL-pol** and **M-dCouAzoC**_**8**_ **-l-CPL-pol** (see Section 3.4 in the SI).

### Chirality Induction in the Supramolecular Complex

The presence of azobenzene moieties in the supramolecular complex allows the chirality in the complex to be induced without the presence of chiral centers. As previously described in the literature, supramolecular chirality can be induced in columnar mesophases by irradiating them with circularly polarized light (CPL).^35–40^ The isomerization of azobenzene, induced by external photoirradiation, alters the polarity and geometry of the compound, resulting in a macroscopic dichroism. Furthermore, if we change the sign of the CPL irradiation to the opposite direction, the obtained circular dichroism (CD) spectrum is the opposite sign, demonstrating that the supramolecular chirality can be externally tuned to the sign of the employed CPL (Figure 3e).

The CD signal is the result of a helical arrangement of the chromophores through the columns of the material, which can be modulated with the sign of the CPL. The presence of the π–π* shoulder at 375 nm in the UV spectra confirmed the trans state of the azo moieties (see Figure S16 in Section 3.5 of the SI). The signal obtained is independent of the rotation angle and is maintained after 24 h of storing the sample in the dark (see Figure S17 in Section 3.5 of the SI).

The hexagonal columnar arrangement and the chiral order generated in the structure were maintained after coumarin photodimerization imposed by exposure to light of 325 nm wavelength, yielding polymeric materials **M-dCouAzoC**_**8**_ **-r-CPL-pol** and **M-dCouAzoC**_**8**_ **-l-CPL-pol**. This process induces a [2 + 2] dimerization in the coumarins, generating a cyclobutane ring. This reaction was followed by UV–Vis spectroscopy, observing a decrease in the intensity of the π–π* band at 325 nm of the coumarins due to the disappearance of the double bond of the coumarin ring, until the signal remained constant (Figure 4a). In Figure S18 in Section 3.5 of the SI, a plot of absorption versus irradiation time is collected showing that after 1 h a high degree of photodimerization has already been reached. The CD spectrum was recorded after photo-cross-linking to confirm the retention of chirality in the film, observing an intense chiral signal at the same wavelength as before polymerization (see Figure S19 in Section 3.5 of the SI). The material obtained does not melt and decomposes at high temperatures (T_2%_[°C]>200) when heated (see Figure S20 in Section 3.5 of the SI). Furthermore, it is insoluble in common organic solvents such as DCM, chloroform, or ethanol.

**Figure 4:**
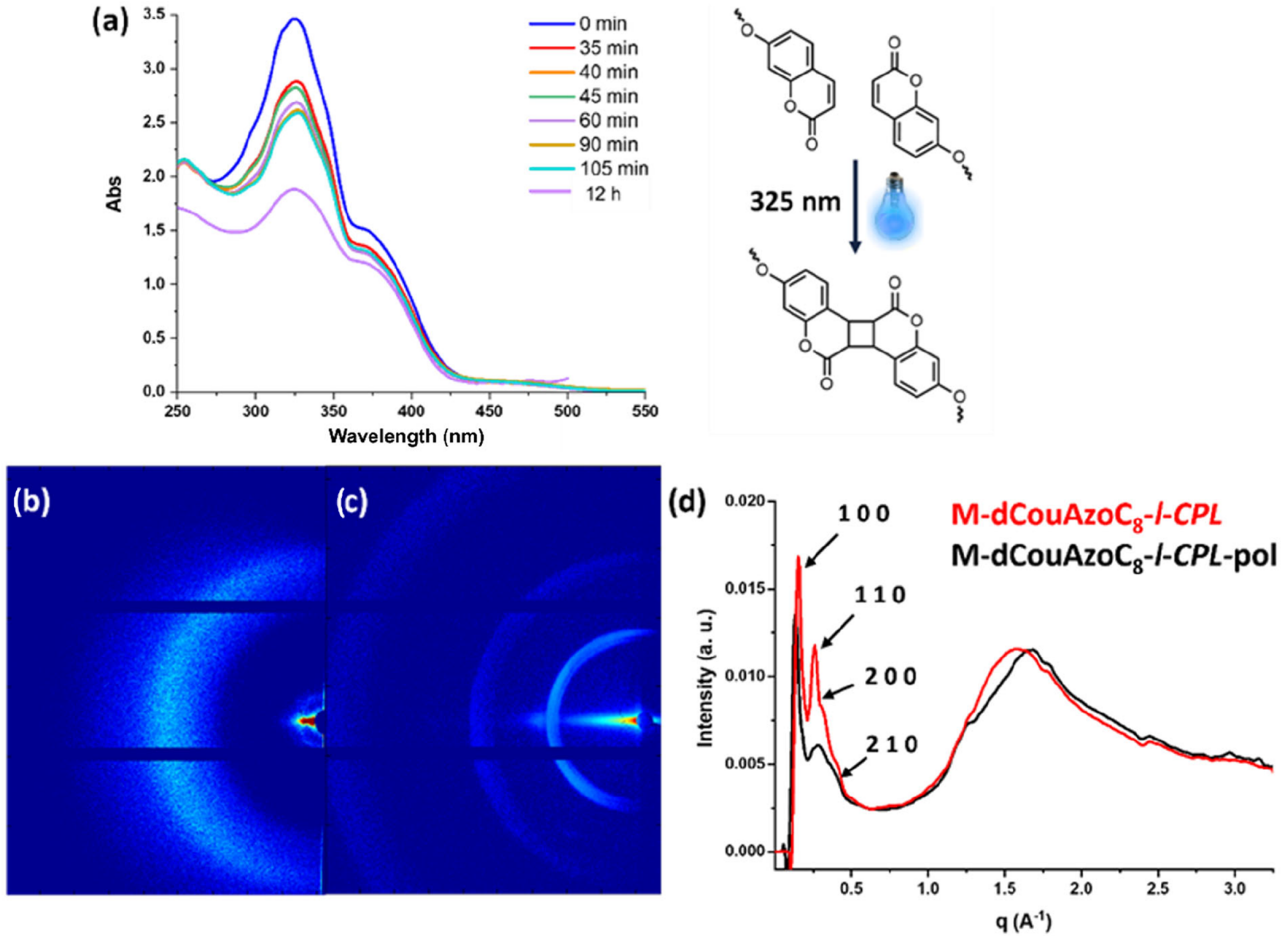
(a) **M-dCouAzoC**_**8**_ **-** ***l-CPL*** photodimerization process followed by UV in film at different times, (b) 2D WAXS pattern after photodimerization (c) 2D MAXS pattern, and (d) 1D XRD comparison of M-dCouAzoC_8_-*l-CPL* before (red line) and after photodimerization (black line) M-dCouAzoC_8_-l-CPL-pol.

The characterization of the polymeric material was carried out using FTIR, observing an appreciable decrease in the intensity of the C=C bond stretching band (which appears at 1613 cm^–1^), confirming the dimerization process (see Figure S21 in Section 3.5 of the SI). The XRD diffraction studies show that the structure maintains the hexagonal columnar order. The XRD diffractograms showed reflections in the (100) and (110) planes, indicating the retention of the hexagonal columnar arrangement in the polymer films with homeotropic alignment (Figure 4b,c,d). These signals showed broadening and shifting in the small angle region, suggesting that the dimerization of coumarins led to a reduction in the column cross-section area and slightly modified lattice distances. As can be seen in Figure S22 in Section 3.5 of the SI, the dimerization of coumarin maintains the sense of helicity in the pore, although the intensity of the signal is slightly modified.

The last step to generate the nanoporous material (**M-dCouAzoC**_**8**_ **-l-CPL-pol**) is removal of the template (**M**). To do this, the polymerized material was treated with an HCl/EtOH mixture (3M), resulting in a self-standing material (Figure 5a). The removal of melamine leaves the carboxyl groups of the dendrons free, generating an acidic environment on the surface of the pores. The percentage of melamine removal was quantified by X-ray photoelectron spectroscopy (XPS) (Figure 5b). In this study, several scans were completed at different depths of the material to evaluate the proportion of nitrogen in the reduction area corresponding to nitrogen (eV) of the melamine.

**Figure 5:**
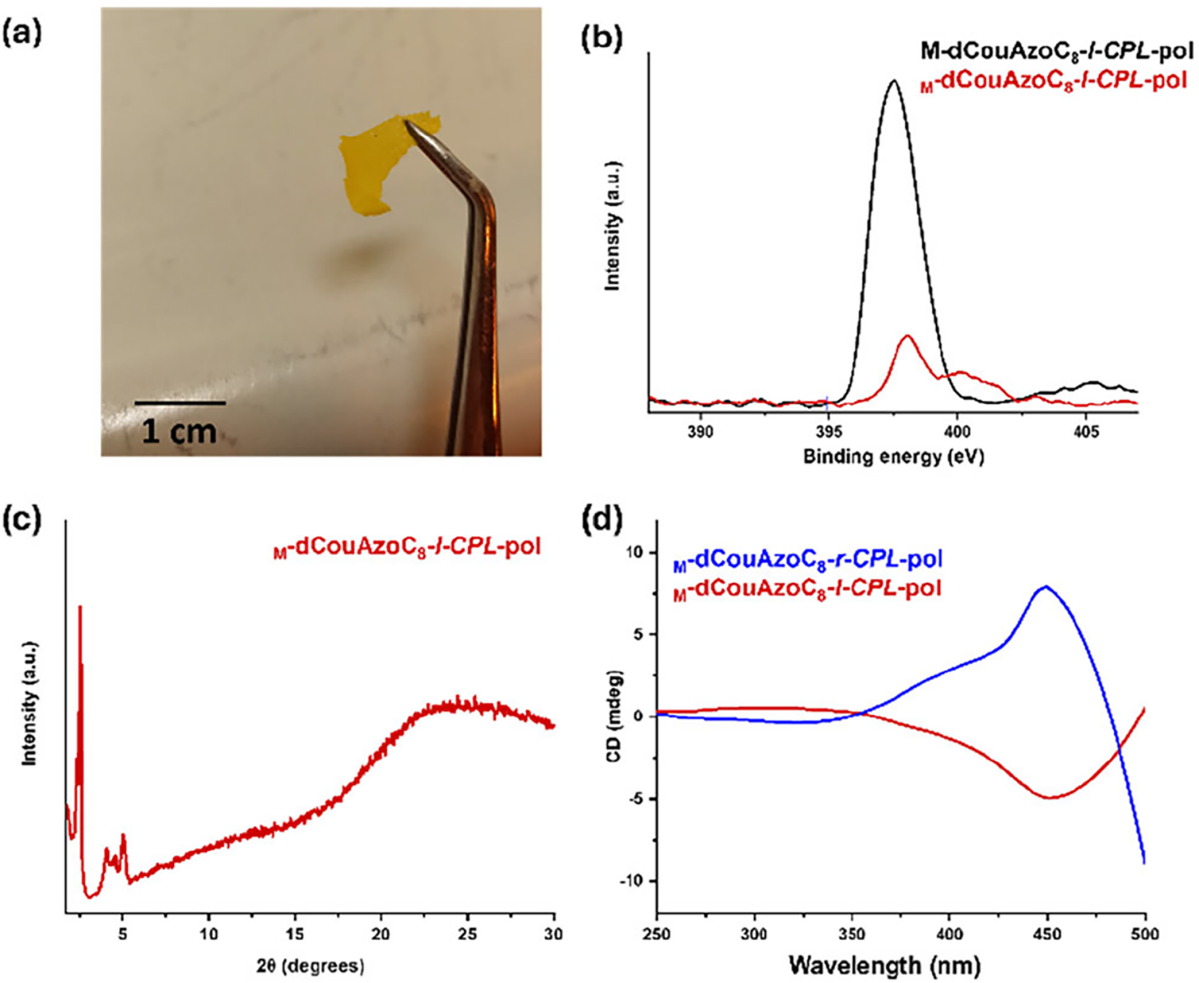
(a) Self-standing _**M**_ **-dCouAzoC**_**8**_ **-l-CPL-pol** nanoporous material. (b) Reduction in the intensity of the nitrogen signal in the nanoporous material _**M**_ **-dCouAzoC**_**8**_ **-l-CPL-pol** in reference to **M-dCouAzoC**_**8**_ **-l-CPL-pol** studied by X-ray photoelectron spectroscopy (XPS). (c) XRD diffractogram of the _**M**_ **-dCouAzoC**_**8**_ **-l-CPL-pol** nanoporous material. (d) CD spectra of the nanoporous material irradiated with right _**M**_ **-dCouAzoC**_**8**_ **-r-CPL-pol** (blue line) or left _**M**_ **-dCouAzoC**_**8**_ **-l-CPL-pol** (red line) circularly polarized light (***CPL***).

The structure of the nanoporous films was characterized by FTIR and XRD. In FTIR, the disappearance of the stretching band of the N–H bonds at 3356 cm^–1^ is observed, which confirms the elimination of melamine. This elimination also produces the appearance of a new band at 1685 cm^–1^ that corresponds to the free carboxylic acid (see Figure S23 in Section 3.5 of the SI). The diffraction patterns observed in XRD are consistent with a two-dimensional (2D) hexagonal lattice, confirming that the chiral nanoporous material maintains the hexagonal columnar arrangement of the original polymer film after template removal (Figure 5c). In this case, the most intense signals correspond to the (100), (200), and (210) planes.

The nanomaterials obtained after removing the template preserve the chiro-optical properties induced by CPL. Thus, Figure 5d shows the circular dichroism (CD) spectrum of the nanoporous material after irradiation with right circularly polarized light (r-CPL), represented by the blue line. To investigate whether the pore chirality could be reversed, the same material was subsequently irradiated with left circularly polarized light (l-CPL). As shown by the red line in Figure 5d, this treatment produced a CD signal of opposite sign, confirming that the chirality of the nanomaterial is reversible and tunable using CPL.

To study the chiral-selective adsorption capacity of the prepared nanoporous materials, the *R,S* enantiomers of (hexan-2-yl)-4-nitroaniline (*R*-NA and *S*-NA) were examined. These molecules have a size similar to the size of the pore generated in the nanoporous materials (approximately 5Å)^23^ and exhibit a basic character that interacts with the acidic environment of the pores. For this study, nanoporous films of opposite chiral sign and similar weight (∼2 mg) were immersed in water solutions of each enantiomer of the same concentration and volume (0.6 mL, 1 × 10^–3^ M). These solutions had been previously studied by UV with a maximum absorbance of 1. The progression of the adsorption of the enantiomers was monitored by UV spectroscopy in the solution at the wavelength of maximum adsorption.

When the solutions of the *R* enantiomer were studied, a small decrease in the UV signal and color intensity was observed in the solutions of both types of films; however, after 2 h, adsorption in the nanopores is not detected anymore in the solutions containing the nanoporous material **M-dCouAzoC**_**8**_ **-r-CPL-pol** (see Figure 6a). On the other hand, in the solutions with the nanoporous material **M-dCouAzoC**_**8**_ **-l-CPL-pol**, the adsorption of the ***R*** enantiomer continues to occur, the adsorption stabilizing after 48 h when most of the ***R*** enantiomer has been absorbed (see Figure 6b). The opposite phenomenon occurs when studying the ***S*** enantiomer. In this case, the enantiomer is almost completely absorbed after 48 h in the solutions containing the **M-dCouAzoC**_**8**_ **-r-CPL-pol** nanoporous material (see Figure 6d), while in the solutions with **dCouAzoC**_**8**_ **-l-CPL-pol**, this adsorption is noticeably lower (see Figure 6e). The adsorption capacity of the **M-dCouAzoC**_**8**_ **-r-CPL-pol** nanoporous material was studied by UV–vis, and a 29% occupation of the carboxyl groups in the pores was found, which represents 0.87 molecules per pore. In relation to the mass of the nanoporous material, the capacity was 56.10 mg/g (see Section 4 in the SI), and the kinetics of this process is adjusted to a first-order kinetic equation (see Figure 6c). The adsorption/desorption capacity study on the nanoporous materials was carried out during three cycles to check its possible recycling. The recovery of the **M-dCouAzoC**_**8**_ **-l-CPL-pol** nanoporous materials containing *S*-(hexan-2-yl)-4-nitroaniline (***S*** **-NA**) was performed by immersing the membrane in KOH/H_2_O (1M) and studying the concentration of carboxyl groups in the pores. The results show that the adsorption–desorption process is reversible and produces only very small decreasing changes in the three cycles studied (Figure 6f).

**Figure 6:**
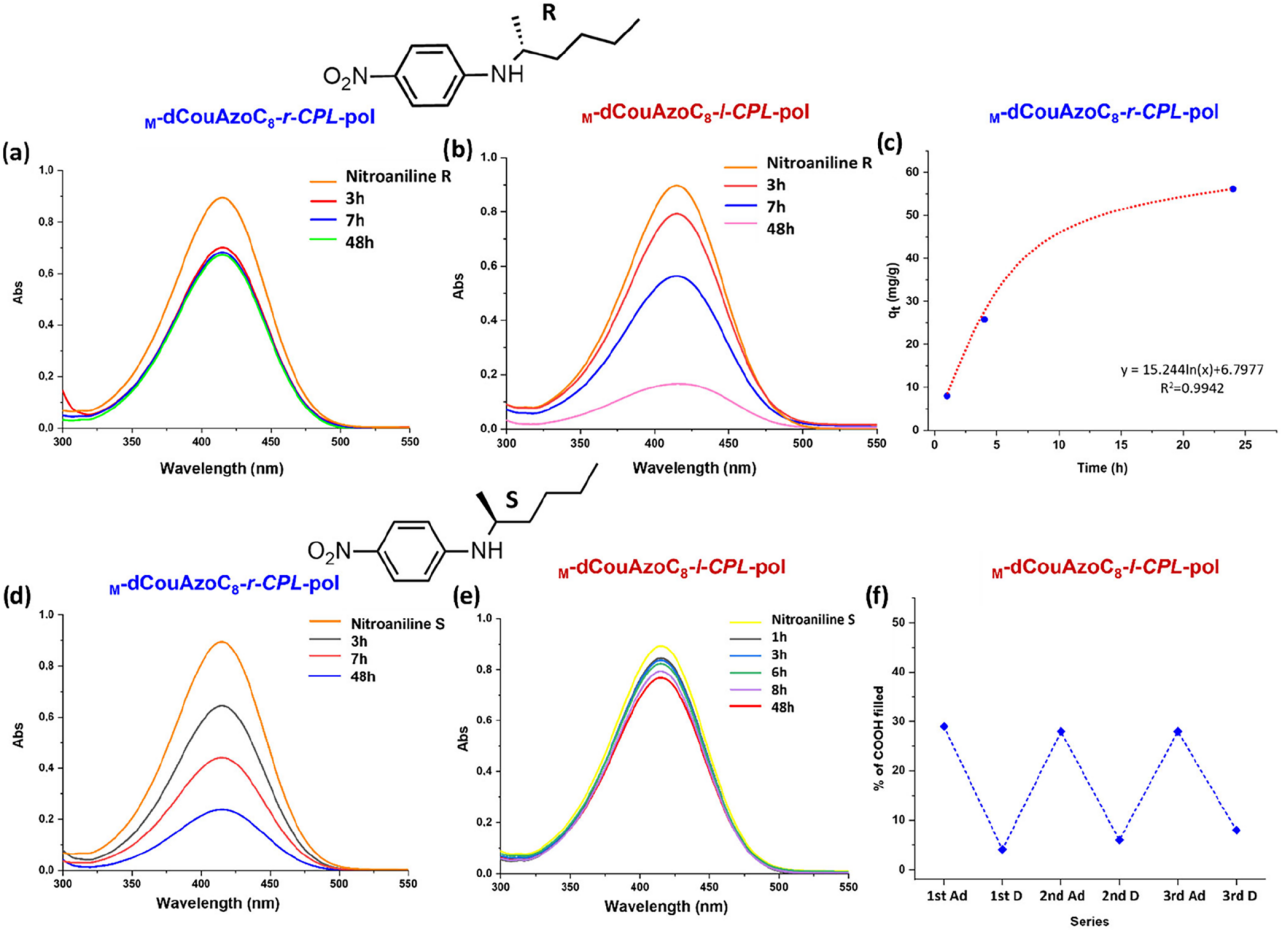
(a, b, d, e) Selective adsorption of the two enantiomers of (hexan-2-yl)-4-nitroaniline in the nanoporous materials _**M**_ **-dCouAzoC**_**8**_ **-r-CPL-pol** and _**M**_ **-dCouAzoC**_**8**_ **-l-CPL-pol.** (c) Adjustment to a first-order kinetic adsorption in the nanoporous material _**M**_ **-dCouAzoC**_**8**_ **-r-CPL-pol**. (f) Three adsorption–desorption cycles of _**M**_ **-dCouAzoC**_**8**_ ***-*** **l-CPL**-**pol**.

Taking into account the chiral nanoporous materials’ capacity to adsorb/reject molecules due to their chirality, we considered their use to separate racemic mixtures. For this purpose, an equimolar solution of both ***S*** **-NA** and ***R*** **-NA** enantiomers in water (0.6 mL, 2 × 10^–3^ M) was prepared, and **M-dCouAzoC**_**8**_ **-l-CPL-pol** nanoporous material was immersed in it. After 24 h, the concentration of the two enantiomers in the solution was studied by chiral HPLC (high-performance liquid chromatography), showing an enantiomer ratio of 20/80 (***R*** **-NA**)/(***S*** **-NA**), demonstrating the enantioseparation from racemic mixtures (Figure 7a). The inverse **M-dCouAzoC**_**8**_ **-r-CPL-pol** nanoporous material was also investigated, obtaining an opposite 70/30 ratio of the (***R*** **-NA**) to *(* **S-NA**) enantiomers (Figure 7b). These experiments confirm the ability of the chiral nanoporous materials to separate racemic mixtures and their capacity to adsorb the desired enantiomer with the same material irradiated with ***r*** or ***l-CPL***.

**Figure 7:**
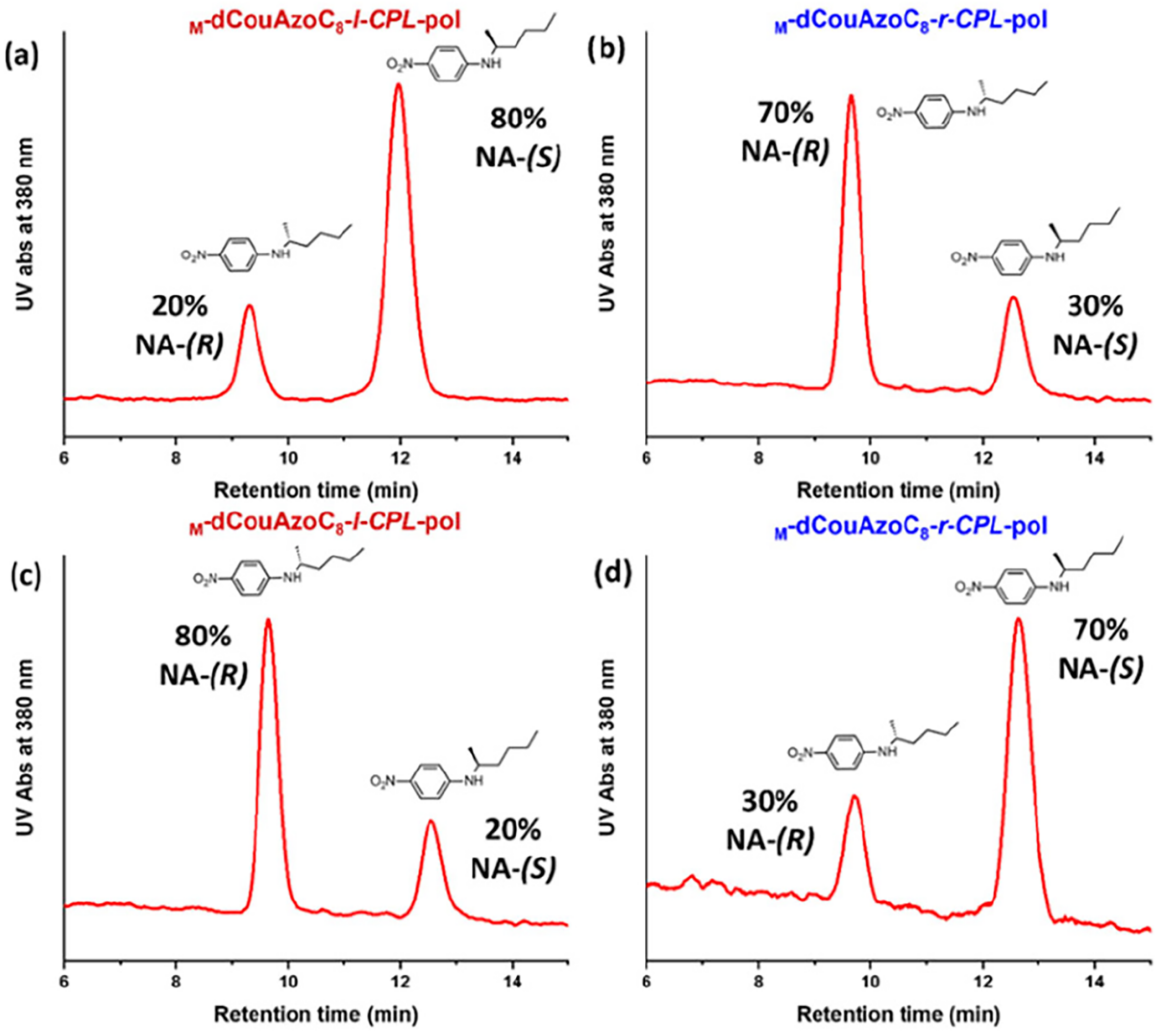
(a) and (b) Ratio of the enantiomers in a racemic solution of ***R*** **-NA** and ***S*** **-NA** (in water 2 × 10^–3^ M) after 24 h of immersion of the nanoporous material _**M**_ **-dCouAzoC**_**8**_ **-l-CPL-pol** and _**M**_ **-dCouAzoC**_**8**_ **-r-CPL-pol**, respectively. (c) and (d) Ratio of the enantiomers ***R*** **-NA** and ***S*** **-NA** in a KOH/H_2_O (1M) solution after 24 h of immersion of the nanoporous materials obtained in the previous processes **a** and **b**.

Finally, both nanoporous materials obtained in the previous process were immersed in a KOH/H_2_O (1M) solution. After 24 h, the solutions turned yellow and were analyzed by chiral HPLC, resulting in the opposite concentration of enantiomers to that obtained in the previous solutions (Figure 7c,d). This confirms that the adsorption–desorption process is reversible, and the chiral compounds adsorbed in the nanoporous materials are released into the solution.

## Conclusions

In this work, we have successfully demonstrated the preparation of chiral nanoporous materials. The unique design is based on a disk-like supramolecule formed by a melamine derivative, which acts as a template and interacts through hydrogen bonds with three dendrons functionalized with an azobenzene group and two coumarin terminal groups. These materials exhibit a homeotropic columnar arrangement, and due to the azobenzene group, controllable chirality is induced when irradiated with circularly polarized light (CPL).

Photodimerization of the coumarin groups allows us to obtain a mechanically stable film. Finally, removal of the melamine derivative, which acts as a template, and irradiation with circularly polarized light produce a material with chiral nanopores that maintain the homeotropic hexagonal columnar organization. These materials allow to absorb predominantly one of the *R*,*S* enantiomers of (hexan-2-yl)-4-nitroaniline based on their porous chirality. In addition, the chirality of the pores can be reversed by changing the handedness of the CPL, allowing the same material to be used for the enrichment of solutions of racemic mixtures in a given enantiomer. In conclusion, this work provides a new approach to a versatile platform for enantioselective separation with the advantage of external control over chirality.

## Supplementary Material



## Notes

Experimental details; synthetic procedures; structural characterization data including NMR spectra; UV–Vis–IR-FT absorption spectra; circular dichroism spectra; MALDI-TOF mass spectra; XPS spectra; and chiral HPLC analysis and thermal and thermodynamic characterization including POM studies; TGA and DSC studies and X-ray diffraction; and other additional information (PDF)

## ⊥

Department of Chemical Engineering and Chemistry Eindhoven University of Technology Groene Loper 3, Eindhoven 5612AE, The Netherlands
