# Supplementary material for: Phototunable Chiral-Selective Nanoporous Material
Source: ACS Appl Mater Interfaces. 2025 Oct 21;17(44):61007–17. doi: 10.1021/acsami.5c14743 (PMC12598689; doi:10.1021/acsami.5c14743)
Supplement: Supplementary file 1 [file am5c14743_si_001.pdf]

Supporting Information

Phototunable Chiral-Selective Nanoporous Material

Iván Marín[a,b,c], Pilar López Ram de Viu[b,d], Pilar Romero[a,b], Danqing Liu[e], Dirk J. Broer[e], Joaquín Barberá[a,b], and José Luis Serrano[a,b,*]

[a] Instituto de Nanociencia y Materiales de Aragón (INMA), CSIC-Universidad de Zaragoza, 50009 Zaragoza, Spain.
[b] Departamento de Química Orgánica, Facultad de Ciencias, Universidad de Zaragoza, 50009 Zaragoza, Spain.
[c] Present address: Department of Chemical Engineering and Chemistry Eindhoven University of Technology Groene Loper 3, Eindhoven 5612AE, The Netherlands.
[d] Instituto de Síntesis Química y Catálisis Homogénea (ISQCH), CSIC-Universidad de Zaragoza, 50009 Zaragoza, Spain
[e] Department of Chemical Engineering and Chemistry Eindhoven University of Technology Groene Loper 3, Eindhoven 5612AE, The Netherlands.

** Author for correspondence: joseluis@unizar.es*

Contents

1. Synthetic Procedures 3

1.1. Synthesis of N^2-dodecyl-1,3,5-triazine-2,4,6-triamine (N-dodecylmelamine) (M). 3

1.2. Synthesis of lateral dendron dCouAzoC$_8$ 5

1.2.1. Synthesis of (*E*)-1-(4-(2-bromoethoxy)phenyl)-2-(4-(octyloxy)phenyl)diazene) (AzoC$_8$). 8

1.2.2. Synthesis of coumarin derivative 7-(11-bromoundecyloxy)-2H-chromen-2-one (CouC$_{11}$Br).

1.2.3. Synthesis of the lateral dendron dCouAzoC8 (7). 10

2. Preparation and characterization of the Supramolecular Complex M-dCouAzoC$_8$. 14

2.1 Synthesis and characterization. 14

2.2. The continuous variation method applied to ^{1}H NMR experiments on the complex M-dCouAzoC$_8$. 17

2.3. POM microphotographs of M-dCouAzoC$_8$. 18

2.4. Calculation of the stoichiometry of the complex based on the X-Ray structural parameters. 19

3. Preparation and Characterization of the Chiral Nanoporous Materials: M-dCouAzoC$_8$-*r-CPL*-pol and M-dCouAzoC$_8$-*l-CPL*-pol. 20

3.1. Scheme S5. 20

3.2 Irradiation of M-dCouAzoC$_8$ in the columnar hexagonal phase with circular polarized phase to induce the helical organization in the column yielding the complexes M-dCouAzoC$_8$-r-CPL and M-dCouAzoC$_8$-l-CPL. 20

3.3 Preparation of the polymeric structures derived by M-dCouAzoC$_8$-r-CPL and M-dCouAzoC$_8$-l-CPL for photodimerization of the coumarin units: M-dCouAzoC$_8$-r-CPL-pol and M-dCouAzoC$_8$-l-CPL-pol. 20

3.4 Preparation of the nanoporous material by removal of the template molecules in the polymeric precursors: M-dCouAzoC$_8$-*r-CPL*-pol and M-dCouAzoC$_8$-*l-CPL*-pol. 20

3.5. Complementary figures 21

4. Adsorption Capacity of the Chiral Nanoporous Materials and Kinetics of the Process. 25

1. Synthetic Procedures

1.1. Synthesis of the *N*²-dodecyl-1,3,5-triazine-2,4,6-triamine (N-dodecylmelamine) (M)

The synthetic route for the preparation of the **N-dodecylmelamine (M)** is shown in ***Scheme 1***.

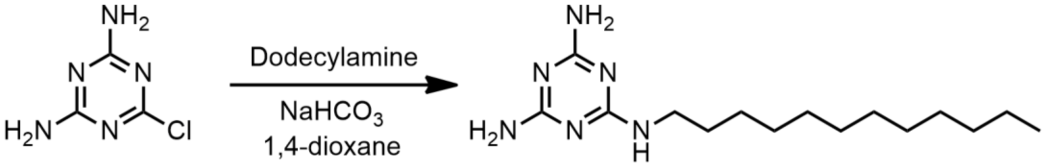

Scheme S1. *Synthetic route of* ***N-dodecylmelamine (M).***

Synthesis of N-dodecylmelamine: A mixture of 2,4-diamino-6-chloro-1,3,5-triazine (2.77 g, 19.0 mmol), dodecylamine (3.52 g, 19.0 mmol) and sodium hydrogen carbonate (1.60 g, 19.0 mmol) was dissolved in 1,4-dioxane (75 ml). The mixture was stirred under argon atmosphere for 6h at reflux. The reaction mixture was poured into water and the precipitate was filtered off and washed with water. The crude was purified by flash column chromatography on silica gel using DCM/MeOH (10:1) as eluent. Finally, the product was recrystallized in ethanol with a yield of 53%.

^{1}H-NMR (CD_2Cl_2, 298K, 400 MHz, δ: ppm): 4.97-4.83 (m, 5H), 3.31-3.27 (m, 2H), 1.53-1.50 (m, 2H), 1.44-1.27 (m, 18H), 0.88 (t, *J*=6.7 Hz, 3H). ***See Figure S1.***

^{13}C-NMR (CD_2Cl_2, 298K, 100 MHz, δ: ppm): 168.21, 167.54, 41.20, 32.50, 30.37, 30.19, 29.95, 27.49, 23.26, 14.43. ***See Figure S2.***

FT-IR (KBr, ν, cm^{-1}): 3495, 3440, 3330 (N-H), 1665 (C=N).

MS (ESI^+, m/z): found 295.26 [$M+H^+$], calculated 294.44.

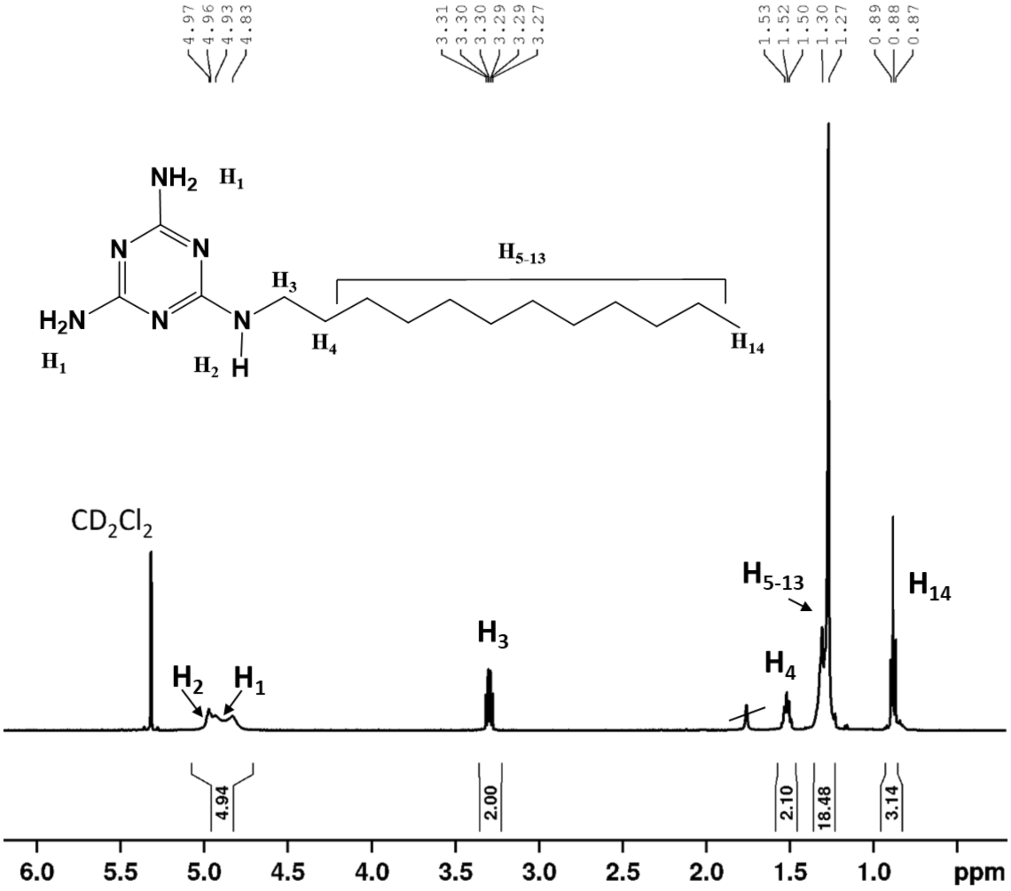

Figure S1. *^{1}H-NMR spectrum of* **N-dodecylmelamine**, *CD_2Cl_2, 298 K, 400 MHz.*

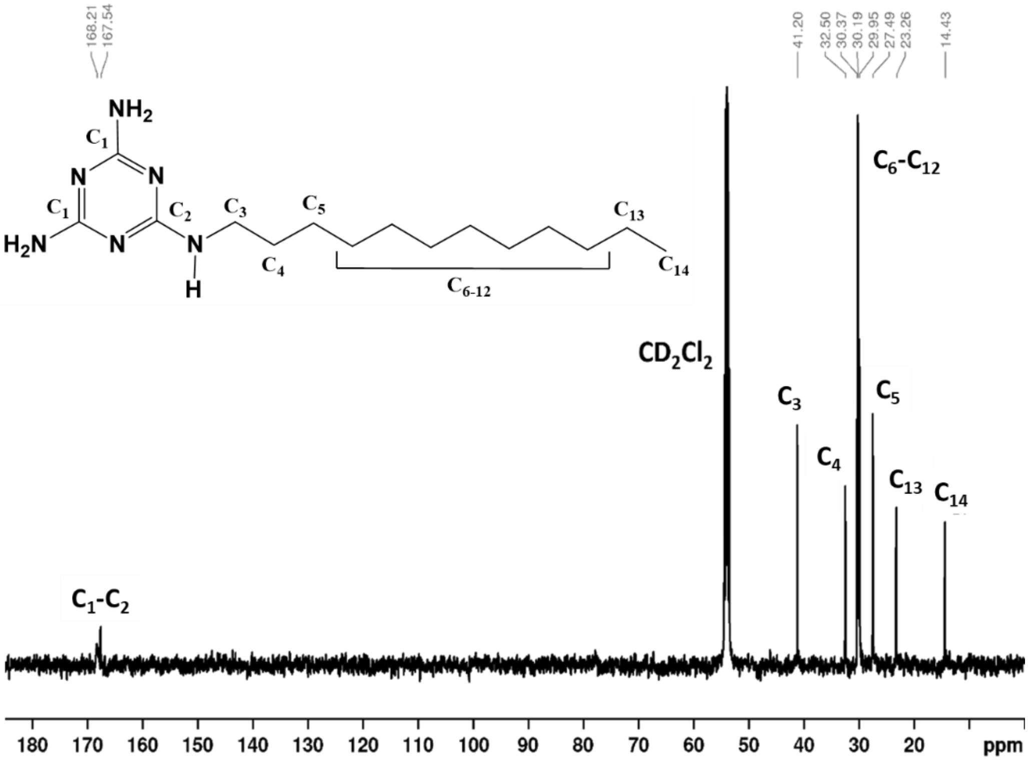

Figure S2. *^{13}C-NMR spectrum of* **N-dodecylmelamine**, *CD_2Cl_2, 298K, 100 MHz.*

1.2. Synthesis of lateral dendron dCouAzoC$_8$

1.2.1. Synthesis of (*E*)-1-(4-(2-bromoethoxy)phenyl)-2-(4-(octyloxy)phenyl)diazene) (AzoC$_8$)

The synthetic route for the preparation of the precursor **AzoC$_8$** is shown in ***Scheme S2***.

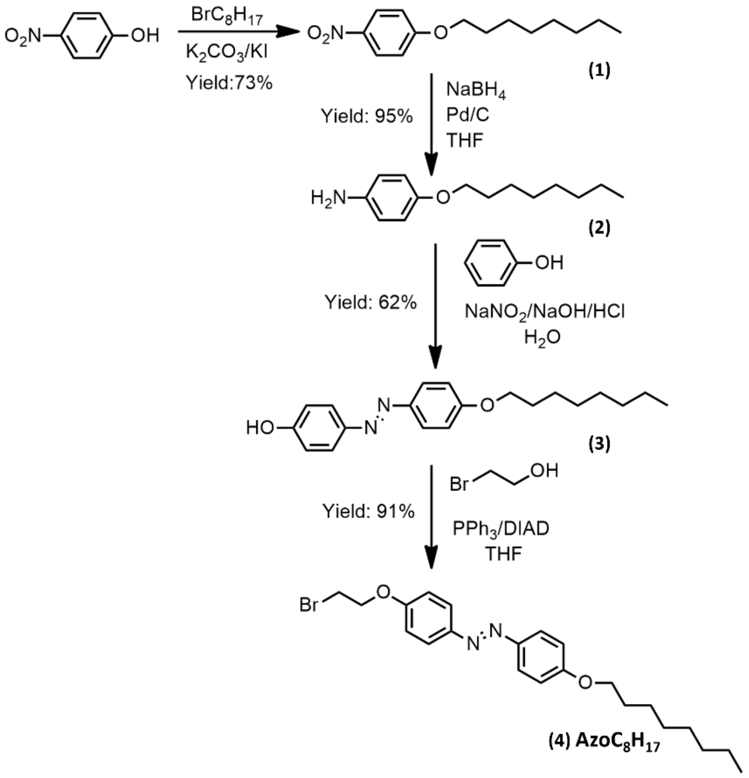

Scheme S2. *Synthetic route for* **AzoC$_8$.**

Synthesis of **1-nitro-4-(octyloxy)benzene (1):** in a flask were dissolved in acetone (100 ml) 4-nitrophenol (10.00 g, 71.94 mmol) and K_2CO_3 (19.86 g, 143.88 mmol). After vigorous stirring during one hour at reflux, 1-bromooctane (20.84 g, 107.91 mmol) and a spatula tip of KI were added. The mixture was stirred under reflux for 10 hours, filtered over celite and the solvent removed. The crude was purified by column chromatography in an eluent mixture of dichloromethane 1:1 hexane with a yield of 73%.

^{1}H-NMR (CDCl$_3$, 298K, 400MHz, δ: ppm): 8.20-8.16 (m, 2H), 6.95-6.90 (m, 2H), 4.04 (t, J=6.5 Hz, 2H), 1.82 (m, 2H), 1.50-1.23 (m, 10H), 0.89 (t, J=7.1 Hz, 3H)

^{13}C-NMR (CDCl$_3$, 298K, 100MHz, δ: ppm): 164.40, 141.42, 126.03, 114.52, 69.04, 31.91, 29.40, 29.32, 29.10, 26.04, 22.77, 14.21.

Synthesis of **4-(octyloxy)aniline (2):** In a flask was prepared a solution of 1-nitro-4-(octyloxy)benzene **(1)** (10.00 g, 39.78 mmol) in 100 ml of THF. Under argon atmosphere were added $NaBH_4$ (3.00 g, 79.57 mmol) and 1.00 g of Pd/C, and the mixture was stirred at room temperature for 3 hours. The crude was filtered over celite and the solvent removed, obtaining the product with a yield of 95%.

^{1}H-NMR ($CDCl_3$, 298K, 400 MHz, δ: ppm): 6.76-6.71 (m, 2H), 6.65-6.60 (m, 2H), 3.87 (t, J=6.6 Hz, 2H), 3.40 (s, 2H), 1.74 (m, 2H), 1.50-1.20 (m, 10H), 0.88 (t, J=6.9 Hz, 3H).

^{13}C-NMR ($CDCl_3$, 298K, 100 MHz, δ: ppm): 152.49, 139.94, 116.55, 115.80, 68.86, 31.96, 29.58, 29.53, 29.39, 26.21, 22.80, 14.24.

Synthesis of **HO-AzoC$_8$ (3):** over a solution of 4-(octyloxy)aniline **(2)** (1.30 g, 5.87 mmol) in THF/H_2O/HCl (50 ml/50 ml/10 ml) was slowly added an aqueous solution of $NaNO_2$ (0.77 g, 11.15 mmol) at 0°C. The mixture was stirred 30 minutes at 0°C and a solution of phenol (0.60 g, 6.45 mmol) in aqueous NaOH (2M, 20 ml) was added. The mixture was kept at pH 9-10 for 10 hours at room temperature. After that time, the mixture was neutralized with HCl (10%) obtaining a biphase. The organic phase was extracted, dried with $MgSO_4$, filtered and the solvent removed. The crude was purified by column chromatography in dichloromethane obtaining the product with a yield of 62%.

^{1}H-NMR ($CDCl_3$, 298K, 400 MHz, δ: ppm): 7.87-7.80 (m, 4H), 7.00-6.91 (m, 4H), 5.11 (s, 1H), 4.03 (t, *J*=6.5 Hz, 2H), 1.85-1.78 (m, 2H), 1.52-1.24 (m, 10H), 0.89 (t, *J*=7.0 Hz, 3H).

^{13}C-NMR ($CDCl_3$, 298K, 100 MHz, δ: ppm): 161.37, 158.13, 147.28, 146.99, 124.66, 124.47, 115.89, 114.82, 68.49, 31.95, 31.09, 29.49, 29.36, 26.17, 22.80, 14.24.

Synthesis of **AzoC$_8$ (4):** in a flask were dissolved **HO-AzoC$_8$ (3)** (1.00 g, 3.06 mmol), triphenylphosphine (1.00 g, 3.83 mmol) and 2-bromoethanol (0.48 g, 3.83 mmol) in THF (40 ml) under argon atmosphere. Finally, was slowly added DIAD (0.77 g, 3.83 mmol) and let react 24 hours, the salts were filtered off and the solvent removed. The product was purified by column chromatography in an eluent mixture of dichloromethane:hexane 6:4 with a yield of 91%.

^{1}H-NMR ($CDCl_3$, 298K, 400 MHz, δ: ppm): 7.89-7.85 (m, 4H), 7.02-6.98 (m, 4H), 4.36 (t, J=6.3 Hz, 2H), 4.03 (t, *J*=6.6 Hz, 2H), 3.67 (t, *J*=6.3 Hz, 2H), 1.84-1.80 (m, 2H), 1.50-1.23 (m, 10H), 0.90 (t, *J*=7.0 Hz, 3H) ***See Figure S3.***

^{13}C-NMR ($CDCl_3$ 298K, 100 MHz, δ: ppm): 161.49, 160.03, 147.68, 146.99, 124.55, 124.49, 115.02, 114.82, 68.49, 68.14, 31.95, 29.49, 29.37, 29.35, 28.95, 26.17, 22.80, 14.24. ***See Figure S4.***

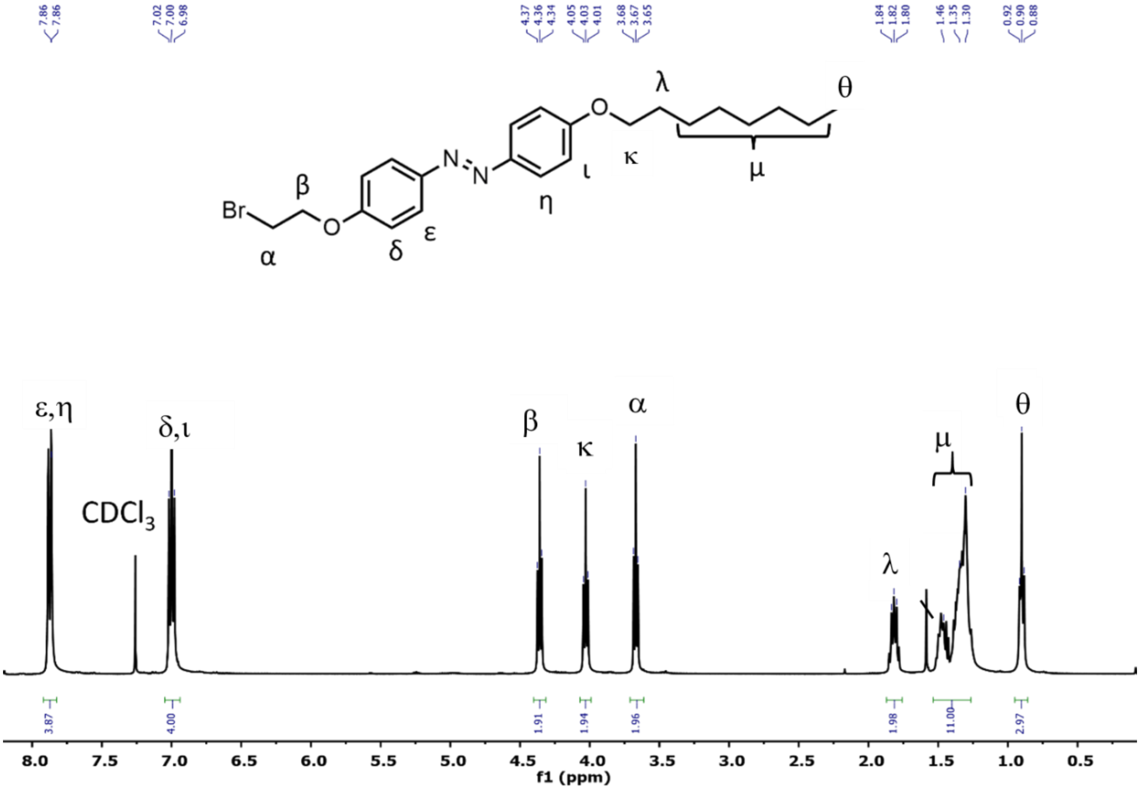

Figure S3. *^{1}H-NMR spectrum of* **AzoC₈**, *$CDCl_3$, 298K, 400 MHz.*

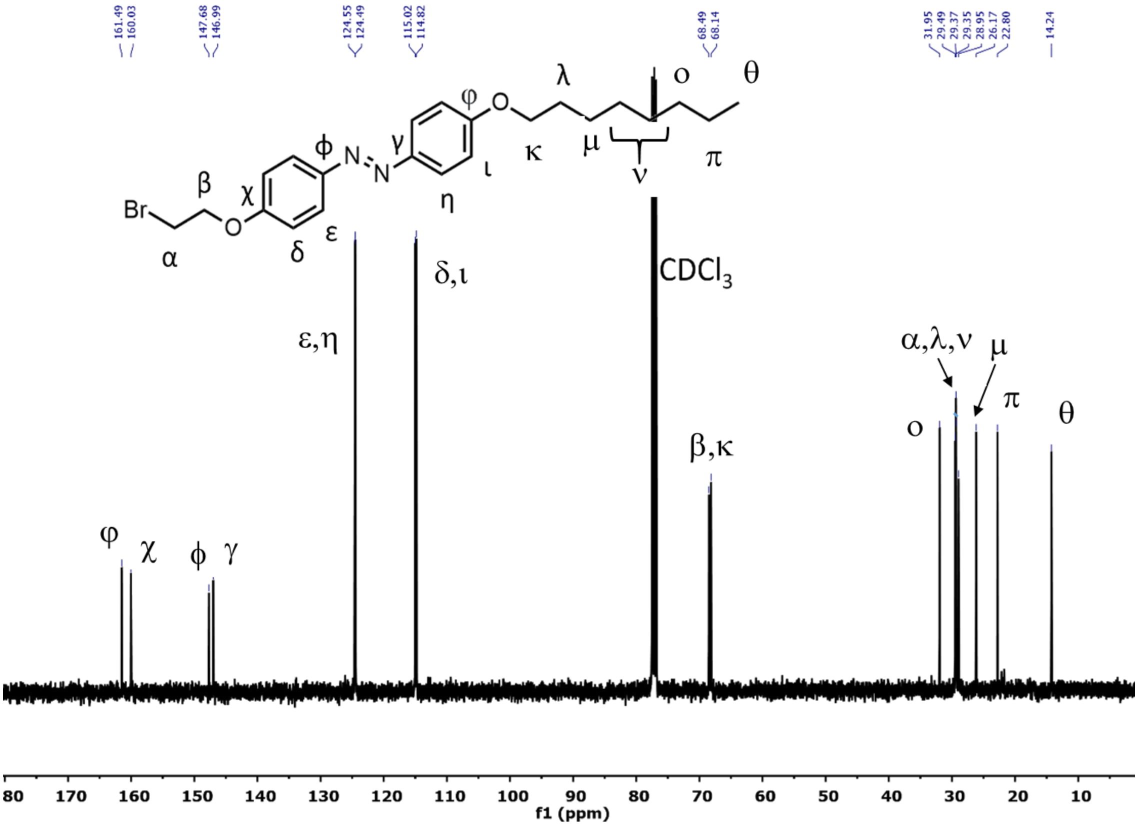

Figure S4. *^{13}C-NMR spectrum of* **AzoC₈**, *$CDCl_3$, 298K, 100 MHz.*

1.2.2. Synthesis of coumarin derivative 7-(11-bromoundecyloxy)-2H-chromen-2-one ($CouC_{11}Br$)

The synthetic route for the preparation of **7-(11-bromoundecyloxy)-2H-chromen-2-one (8)** is shown in ***Scheme S3***.

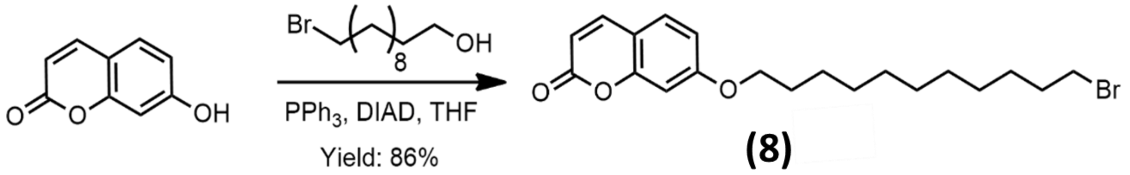

Scheme S3. Synthesis of **7-(11-bromoundecyloxy)-2H-chromen-2-one (8).**

Umbelliferone (8.00 g, 49.34 mmol), 11-bromoundecan-1-ol (12.39 g, 49.34 mmol) and triphenylphosphine (12.94 g, 49.34 mmol) were dissolved in anhydrous THF (450 ml). The reaction flask was cooled in an ice bath and flushed with argon, and then DIAD (9.98 g, 49.34 mmol) was added dropwise. The mixture was stirred at room temperature overnight under an argon atmosphere. The white precipitate was filtered off. The solvent was evaporated and the crude product was recrystallized in ethanol. Yield 86%.

^{1}H-NMR ($CDCl_3$, 298K, 400 MHz, δ: ppm): 7.65 (d, *J*=9.5 Hz, 1H), 7.39 -7.36 (m, 1H), 6.86-6.77 (m, 2H), 6.26 (d, *J*=9.5 Hz, 1H), 4.03 (t, *J*=6.5 Hz, 2H), 3.42 (t, *J*=6.8 Hz, 2H), 1.90-1.84 (m, 4H), 1.47 ppm (m, 4H), 1.50-1.25 (m, 14H). ***See Figure S5.***

^{13}C-NMR ($CDCl_3$, 298K, 100 MHz, δ: ppm): 162.55, 161.40, 156.06, 143.57, 128.82, 113.11, 113.04, 112.49, 101.44, 68.77, 34.19, 32.94, 29.59, 29.55, 29.42, 29.08, 28.87, 28.28, 26.06. ***See Figure S6.***

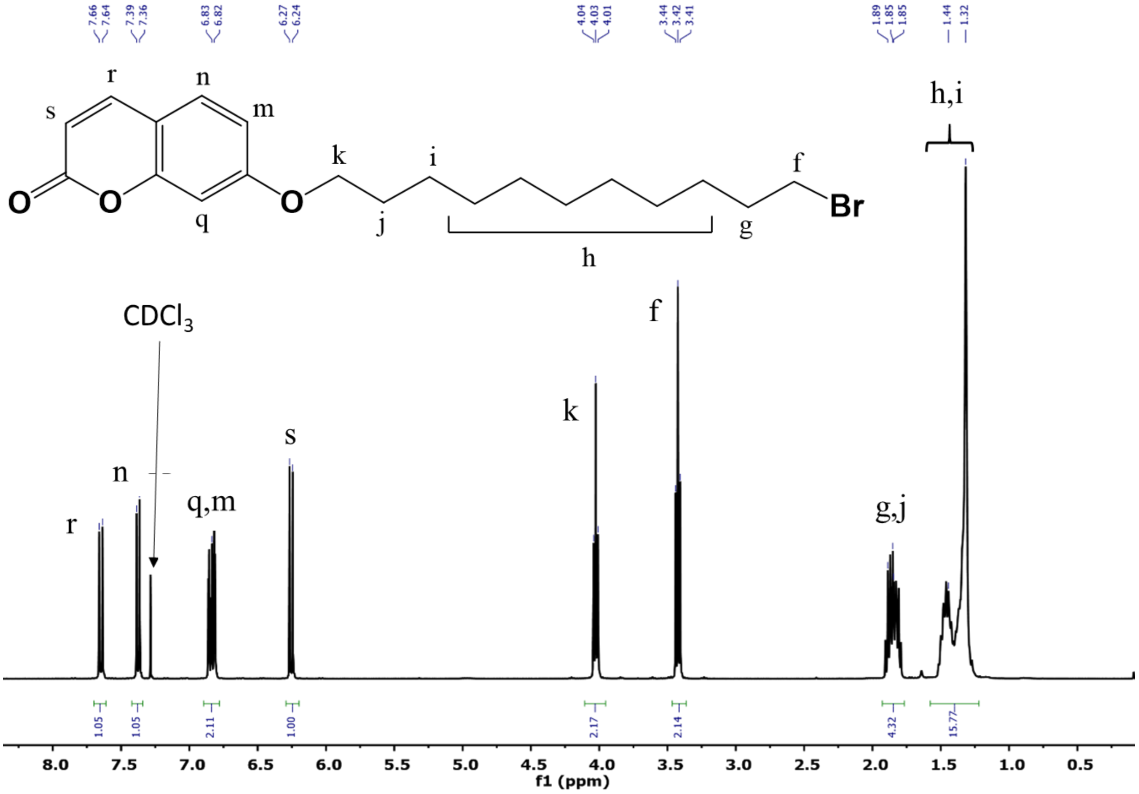

Figure S5. *^{1}H-NMR spectrum of* **CouC11Br**, *$CDCl_3$, 298K, 400 MHz.*

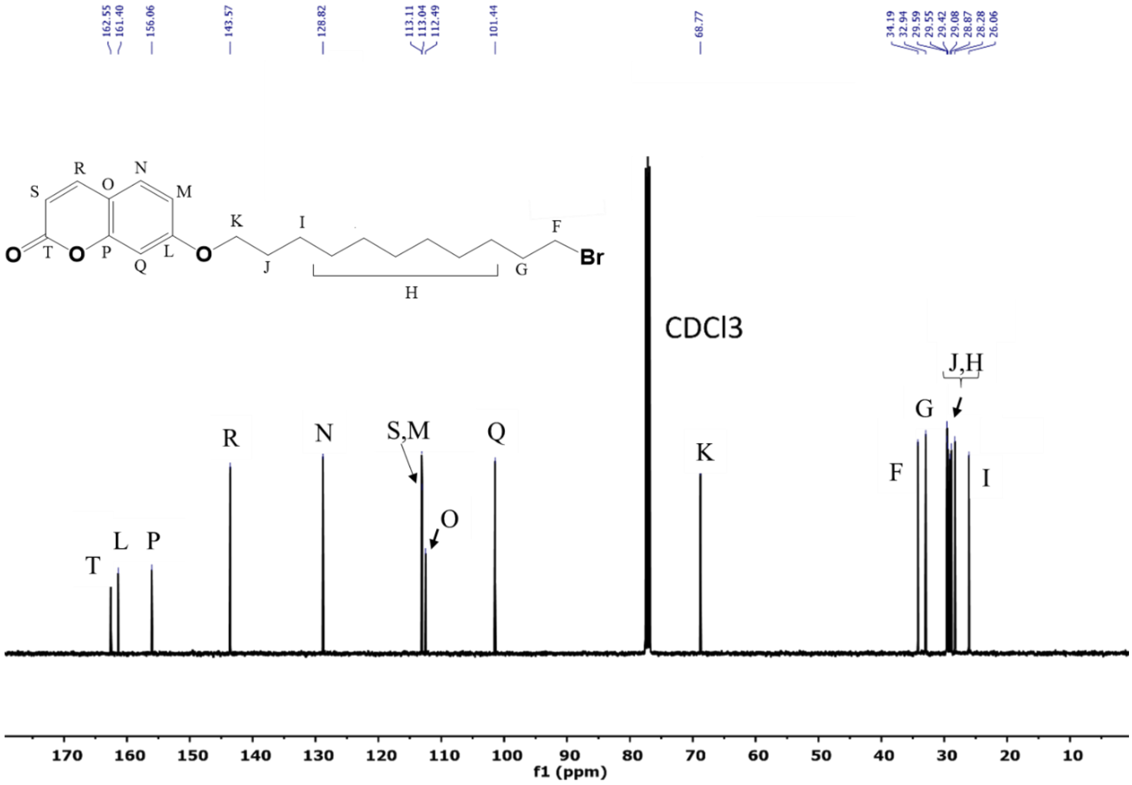

Figure S6. *^{13}C-NMR spectrum of* **CouC11Br**, *$CDCl_3$, 298K, 100 MHz.*

1.2.3. Synthesis of the lateral dendron dCouAzoC$_8$ (7).

The synthetic route for the preparation of the dendronic acid **dCouAzoC$_8$ (7)** is shown in ***Scheme S4***:

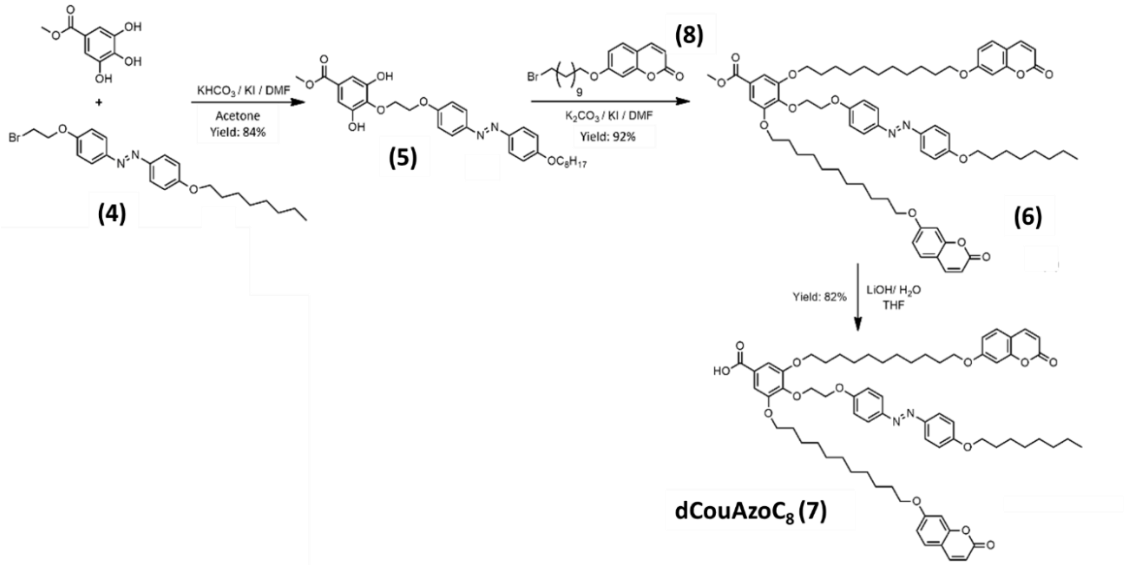

Scheme S4. *Synthesis of the dendronic acid* **dCouAzoC$_8$**.

Synthesis of **intermediate derivative (5)**: in a flask were dissolved methyl gallate (4.25 g, 23.07 mmol), potassium hydrogen carbonate (2.31 g, 23.07 mmol) and a spatula tip of potassium iodide under argon atmosphere in a mixture of DMF/acetone (40/10 ml) at 80°C and it was stirred vigorously. Finally, was added compound **AzoC$_8$ (4)** (1.00 g, 2.31 mmol) and let react overnight. Once the reaction was completed, it was let cool down and the salts were filtered over celite. The solution was dissolved in a mixture of ethyl acetate/hexane (50/50 ml) and extracted with water (5x100 ml), dried with $MgSO_4$, filtered and the solvent removed in vacuum. The crude product was purified by column chromatography on silica gel with an eluent mixture of dichloromethane/hexane (8:2) with a yield of 84%.

^{1}H-NMR (CD_2Cl_2, 298K, 400 MHz, δ: ppm): 7.93-7.85 (m, 4H), 7.19 (s, 2H), 7.14-7.09 (m, 2H), 7.93 -6.89 (m, 2H), 6.34 (s, 2H), 4.48-4.36 (m, 4H), 4.05 (t, *J*=6.6 Hz, 2H), 3.86 (s, 3H), 1.82 (m, 2H), 1.50-1.23 (m,10H), 0.90 (t, *J*=6.9 Hz, 3H).

^{13}C-NMR (CD_2Cl_2, 298K, 100 MHz, δ: ppm): 166.83, 162.19, 160.02, 150.01, 148.47, 147.33, 138.04, 127.75, 124.98, 124.94, 115.43, 115.27, 109.97, 73.31, 69.05, 67.95, 52.63, 32.41, 29.92, 29.82, 29.78, 26.57, 23.25, 14.44.

Synthesis of **intermediate derivative (6):** In a flask were dissolved **(5)** (0.6 g, 1.12 mmol), potassium carbonate (0.46 g, 3.35 mmol) and a spatula tip of potassium iodide in DMF (20 ml) under argon atmosphere. Compound **$CouC_{11}Br$ (8)** (1.10g, 2.79 mmol) was added to the solution and let react at 80°C overnight. The mixture was precipitated in 100 ml of H_2O and extracted with ethyl acetate (3x50 ml); the organic phase was dried with $MgSO_4$, filtered and the solvent evaporated. The crude was purified by column chromatography on silica gel with dichloromethane as an eluent with a yield of 92%.

^{1}H-NMR (CD_2Cl_2, 298K, 400 MHz, δ: ppm): 7.88-7.83 (m, 4H), 7.63 (d, *J*=9.5 Hz, 2H), 7.38-7.33 (m, 2H), 7.27 (s, 2H), 7.02-6.95 (m, 4H), 6.84-6.76 (m, 4H), 6.18 (d, *J*=9.5 Hz, 2H), 4.44-4.40 (m, 2H), 4.35-4.32 (m, 2H), 4.05-3.95 (m, 10H), 3.87 (s, 3H), 1.82-1.73 (m, 10H), 1.50-1.20 (m, 38H), 0.89 (t, *J*=7 Hz, 3H).

^{13}C-NMR (CD_2Cl_2 298K, 100 MHz, δ: ppm): 167.15, 163.02, 161.93, 161.43, 156.55, 153.25, 147.66, 147.37, 143.93, 142.07, 129.32, 125.98, 124.81, 124.79, 115.27, 115.19, 113.37, 113.28, 112.95, 108.07, 101.77, 71.78, 69.75, 69.31, 68.98, 68.62, 52.56, 32.40, 30.13, 30.10, 29.95, 29.92, 29.90, 29.85, 29.82, 29.78, 29.56, 26.61, 26.57, 26.48, 23.24, 14.44.

Synthesis of **$dCouAzoC_8$ (7):** Over a solution of **(6)** (0.40 g, 0.34 mmol) in THF (10 ml) at 60°C, was added a solution of LiOH (41.00 mg, 1.17 mmol) in water (5 ml), and the mixture was stirred overnight. The crude was precipitated in a solution of HCl/H_2O 4M, obtaining an orange solid. The solid obtained was filtered and recrystallized in methanol, obtaining an orange solid with a yield of 82%.

^{1}H-NMR (CD_2Cl_2, 298K, 400 MHz, δ: ppm): 7.86-7.83 (m, 4H), 7.64 (d, *J*=9.5 Hz, 2H), 7.39-7.34 (m, 2H), 7.32 (s, 2H), 7.00-6.96 (m, 4H), 6.80-6.76 (m, 4H), 6.18 (d, *J*=9.5 Hz, 2H), 4.46-4.42 (m, 2H), 4.37-4.33 (m, 2H), 4.03-3.97 (m, 10H), 1.83-1.71 (m, 10H), 1.47-1.29 (m, 38H), 0.89 (t, *J*=7 Hz, 3H). ***See Figure S7.***

^{13}C-NMR (CD_2Cl_2, 298K, 100 MHz, δ: ppm): 170.30, 163.05, 161.95, 161.57, 161.42, 156.55, 153.33, 147.69, 147.39, 144.00, 142.84, 129.34, 124.82, 115.29, 115.21, 113.36, 113.33, 112.97, 108.69, 101.80, 71.84, 69.83, 69.34, 69.00, 68.64, 32.41, 30.08, 29.93, 29.88, 29.82, 29.79, 29.55, 26.59, 26.47, 23.24, 14.44. ***See Figure S8.***

FTIR (KBr, ν: cm^{-1}): 3369 (OH), 2921 (-C=C-H), 2854 (C-C-H), 1742 (C=O), 1677 (C=O), 1618 (C=C), 1511 (C=C), 1239 (C-O), 1125 (C-O).

MS (MALDI$^+$, dithranol, m/z): found 1173.5 [M+Na$^+$] calculated 1151.4. ***See Figure S9.***

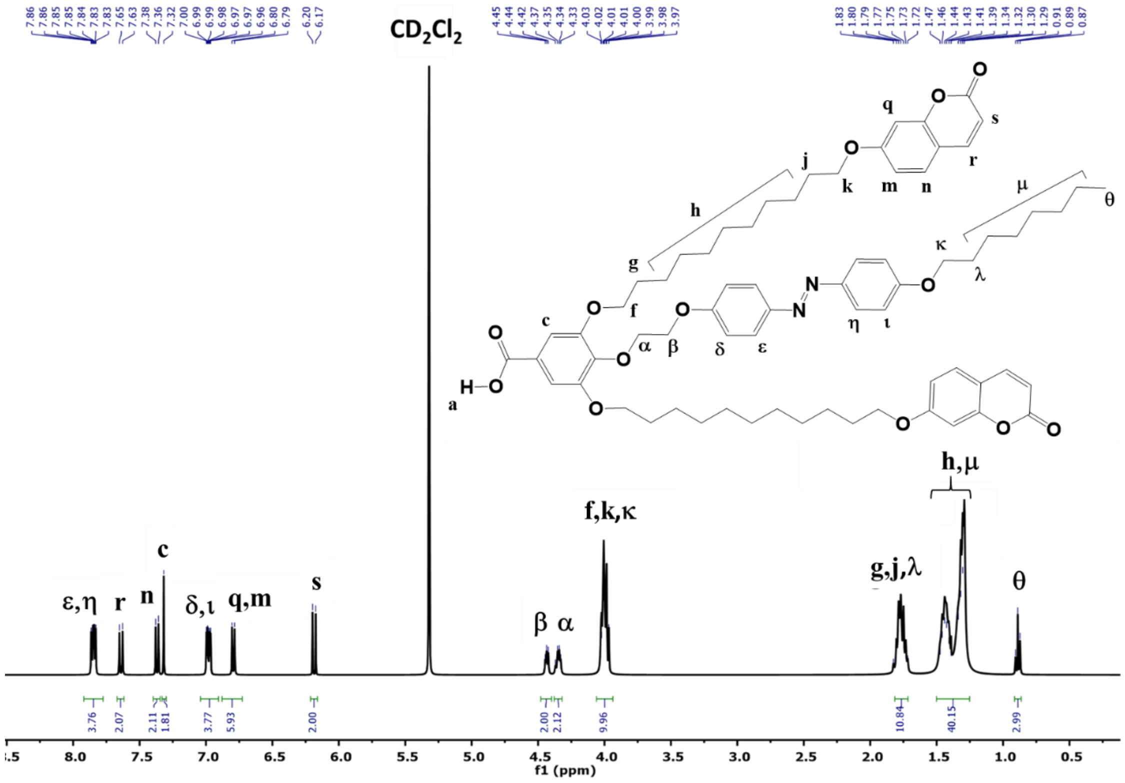

Figure S7. *^{1}H-NMR spectrum of* **dCouAzoC$_8$**, *CD_2Cl_2, 298K, 400 MHz.*

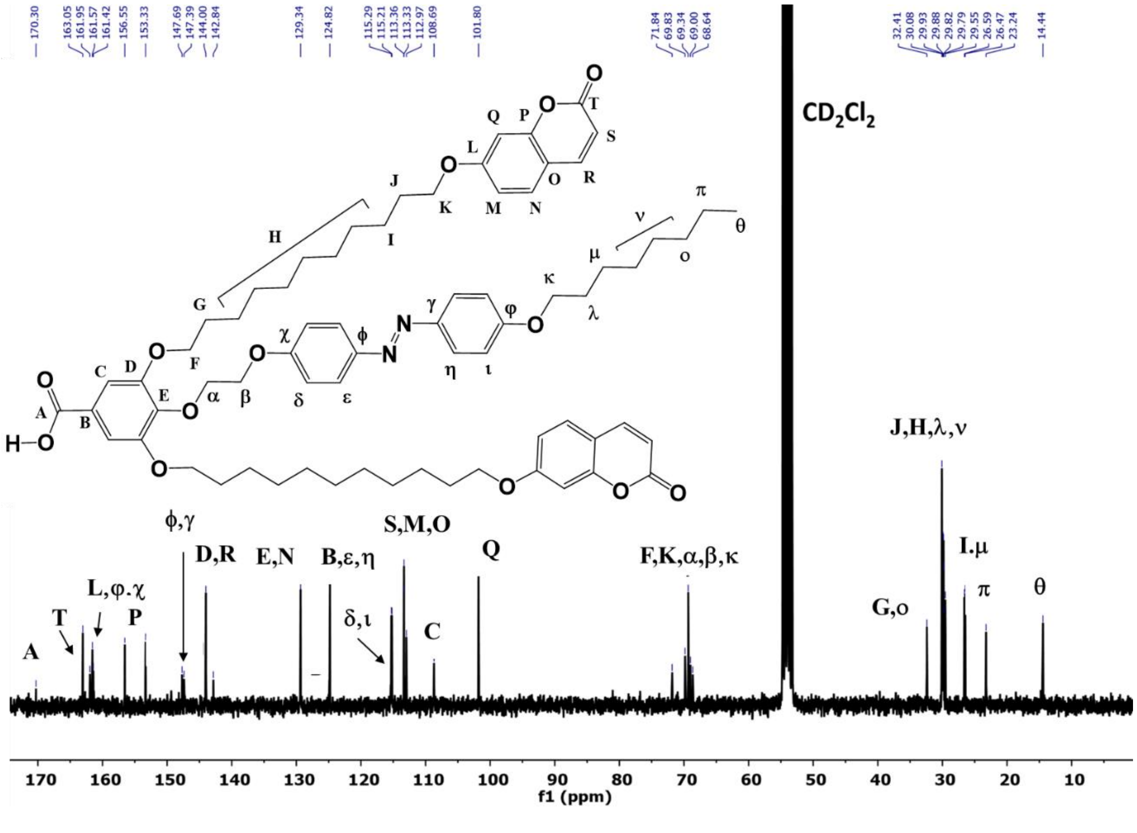

Figure S8. *^{13}C-NMR spectrum of* **dCouAzoC$_8$**, *CD_2Cl_2, 298K, 100 MHz.*

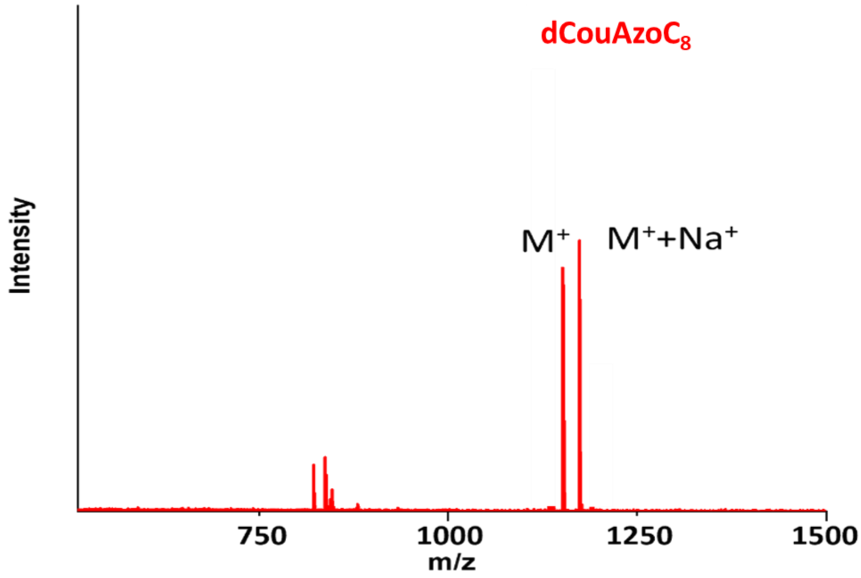

Figure S9. *MALDI spectrum of* **dCouAzoC**$_8$ *(dithranol matrix) found 1173.5 [M+Na*$^+$*]. Calculated data with sodium 1174.38 (1151.4+22.98).*

2. Preparation and characterization of the Supramolecular Complex M-dCouAzoC$_8$.

2.1 Synthesis and characterization

The preparation of the supramolecular complex was carried out by dissolving in dichloromethane (DCM) a mixture in a ratio 1:3 of the melamine derivative (**M**) (bearing a dodecyloxy alkyl chain to favor their solubility), that acts as template core,[40] and three molecular dendrons (**dCouAzoC$_8$**). The solvent was slowly evaporated under continuous stirring at room temperature and dried in vacuum at 40°C until the weight remained constant, yielding the supramolecular complex **M-dCouAzoC$_8$**.

^{1}H-NMR (CD_2Cl_2, 298K, 400 MHz, δ: ppm): 7.88-7.82 (m, 12H), 7.63 (d, J=9.5 Hz, 6H), 7.37-7.35 (m, 6H), 7.32 (s, 6H), 7.01-6.95 (m, 12H), 6.84-6.76 (m, 12H), 6.25 (s, 1H), 6.18 (d, J=9.5 Hz, 6H), 6.01 (s, 2H), 4.45-4.42 (m, 6H), 4.37-4.33 (m, 6H), 4.04-3.95 (m, 30H), 3.39-3.32 (m, 2H), 1.84-1.70 (m, 34H), 1.52-1.21 (m, 140 H), 0.93-0.83 (m, 12H).

^{13}C-NMR (CD_2Cl_2, 298K, 100 MHz, δ: ppm): 170.49, 164.31, 163.05, 161.95, 161.53, 161.45, 156.55, 153.26, 147.68, 147.39, 143.99, 129.34, 126.10, 124.80, 115.29, 115.21, 113.32, 112.97, 108.53, 101.80, 71.81, 69.78, 69.34, 69.00, 68.63, 41.39, 32.51, 32.40, 30.21, 30.09, 29.94, 29.89, 29.83, 29.80, 29.56, 26.62, 26.58, 26.48, 23.24, 14.44. See

Elemental analysis: Theoretical (%): C:71.16, H:7.79, N:4.47, O_2:16.58. Calculated (%): C:70.61, H:7.85, N:4.62, O_2:16.92.

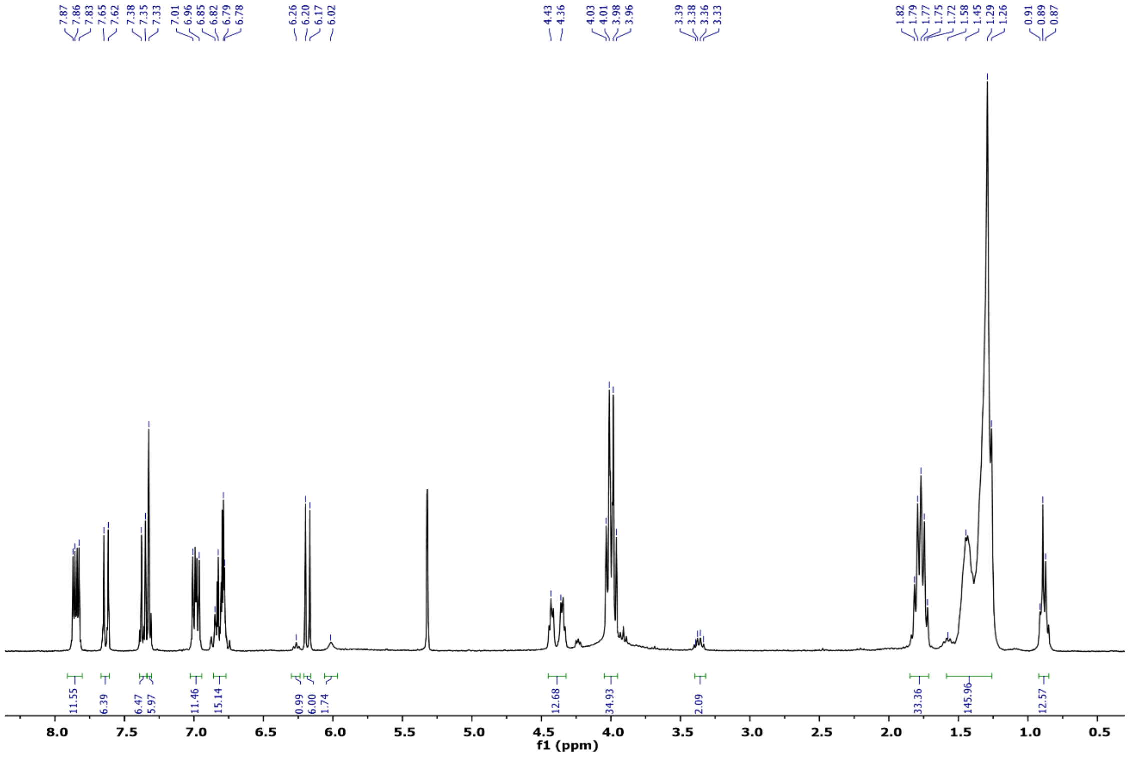

Figure S10. ^{1}H-NMR spectrum of **M-dCouAzoC$_8$**, CD_2Cl_2, 298K, 400 MHz

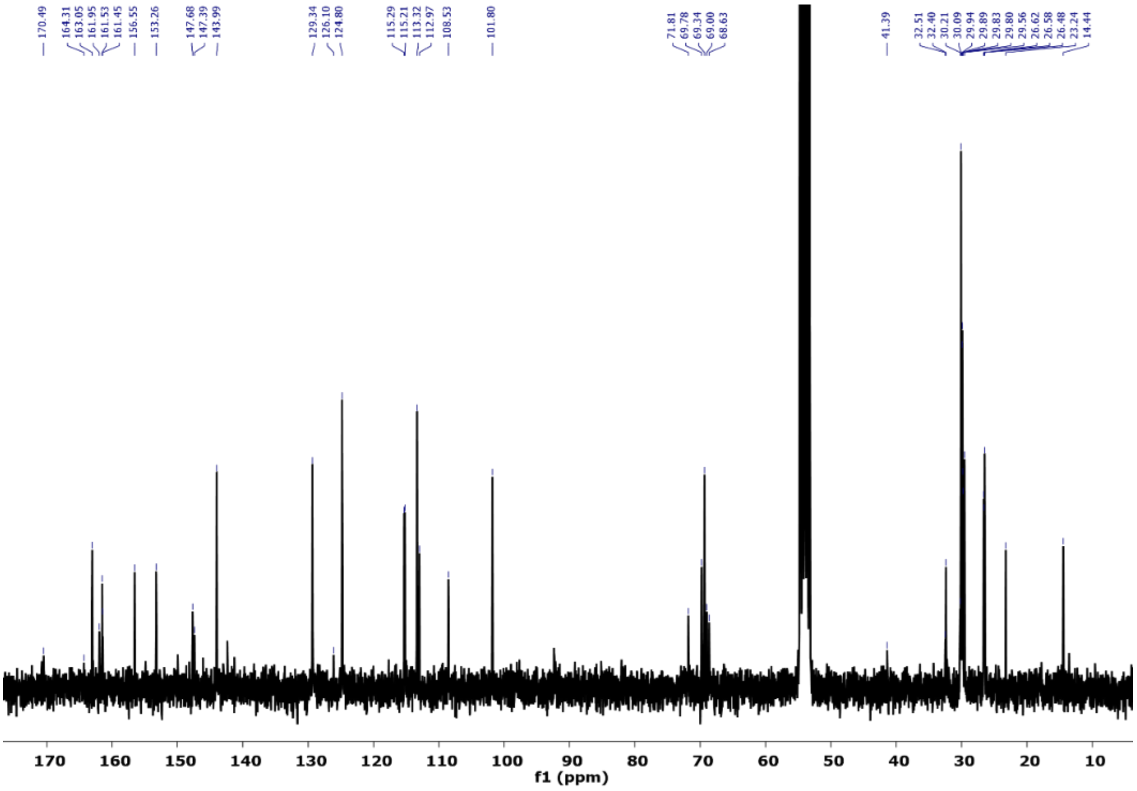

Figure S11. *^{13}C-NMR spectrum of* **M-dCouAzoC$_8$**, *CD_2Cl_2, 298K, 100 MHz.*

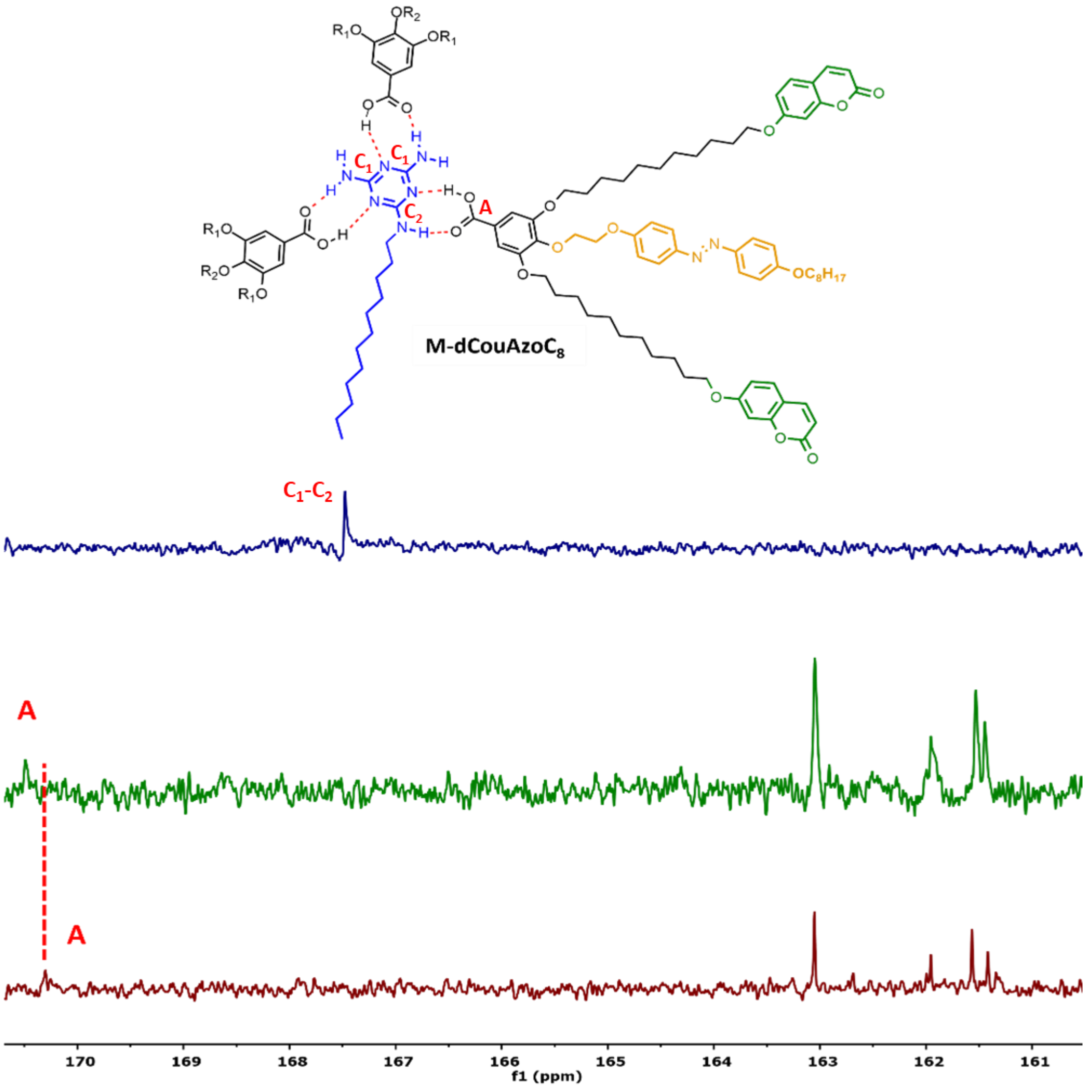

Figure S12. *^{13}C-NMR comparison in CD_2Cl_2 of* **N-dodecylmelamine (M)** *(blue line)* **M-dCouAzoC8** *(green line) and* **dCouAzoC8** *(red line).* ***C_1*** *and* ***C_2*** *carbons of melamine were not detected in the supramolecular complex.*

2.2. The continuous variation method applied to ^{1}H NMR experiments on the complex M-dCouAzoC$_8$.

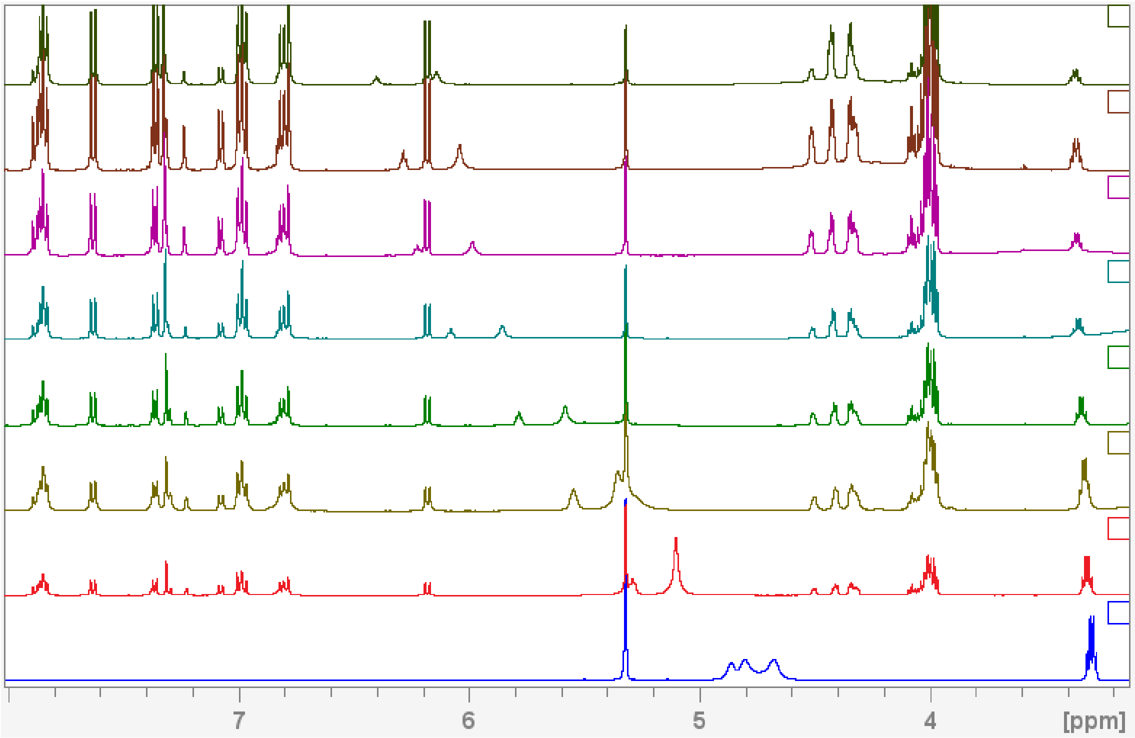

Figure S13. ***^{1}H-NMR*** *spectra obtained for different concentrations of the acid dCouAzoC$_8$, while maintaining constant the concentration of M (1.7 mM in CD_2Cl_2). The acid concentrations are indicated in Table S1.*

Table S1.

[Acid] (M)	δ(NH) (ppm)	δ(CH_2-N) (ppm)
8.47E-04	5.288	3.316
0.00125	5.54	3.328
0.00182	5.781	3.34
0.00228	5.915	3.345
0.0031	6.078	3.354
0.004	6.224	3.361
0.0045	6.283	3.363
0.0061	6.401	3.368

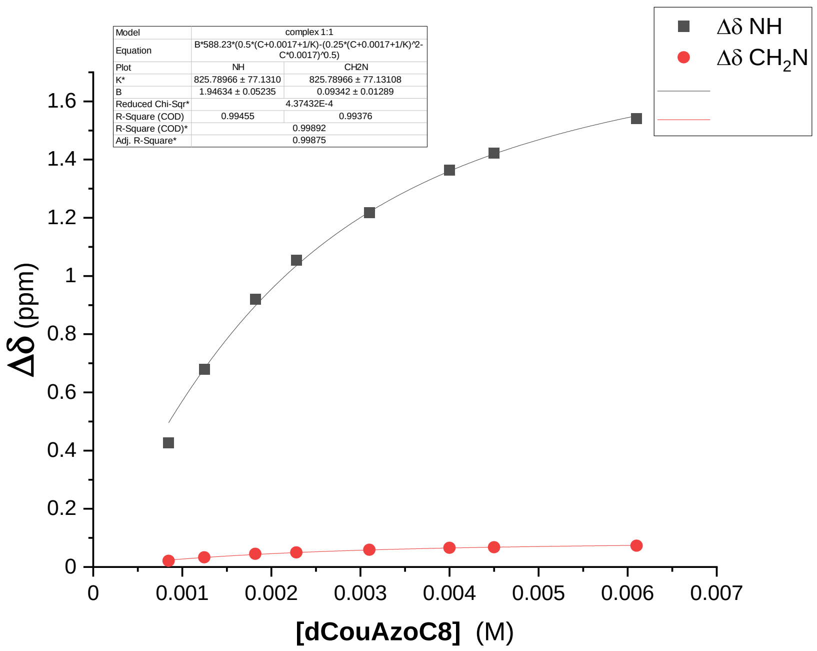

Figure S14. *Concentration dependence of the chemical shifts of* ***NH*** *and* ***CH_2-N*** *in* ***^{1}H-NMR*** *titration experiments.*

2.3. POM microphotographs of M-dCouAzoC_8.

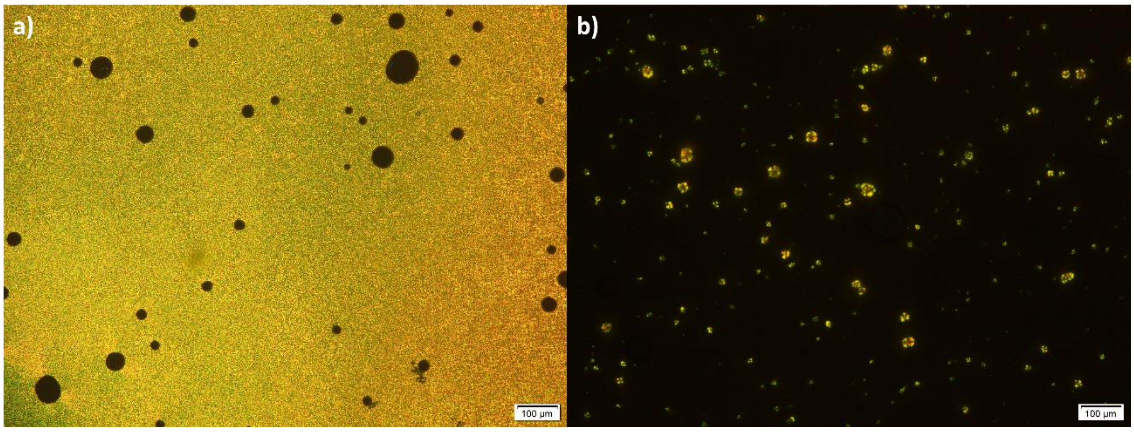

Figure S15. *a) POM microphotograph of* **M-dCouAzoC_8** *at 60°C during the first cooling process and b)* ***POM*** *microphotograph of* **M-dCouAzoC_8** *at 60°C after applying mechanical stress during the first cooling process.*

2.4. Calculation of the stoichiometry of the complex M-dCouAzoC8 based on the X-Ray structural parameters.

Experimental evidence for the stoichiometry 1:3 is deduced from the X-ray diffraction results (Table 1 of the manuscript). Based on the hexagonal columnar stacking of the complexes and the measured structural parameter (hexagonal lattice constant, a = 50.1 Å), some calculations can be performed to obtain an approximate view of the complexes arrangement. The lattice constant **a** (in Å) and the molar mass M of the complex (in g, 3748.6 g for the 1:3 complex) are related to the density ρ (in g cm^{-3}) and the average intermolecular distance h (in Å) along the columnar axis by the following equation:

$$\rho = M \times Z \times 10^{24} / (S \times h \times N_A)$$

where S is the cross-sectional area of the 2D hexagonal lattice (in $Å^2$, $S = (a^2 x\sqrt{3})/2$), a being the hexagonal lattice constant and N_A being Avogadro's number. The deduced cross-sectional area (443.6 $Å^2$) is consistent with the dimensions of a 1:3 complex and not with the dimensions of a 1:2 or any other stoichiometric complex. Furthermore, even more importantly, from the formula mentioned above, although ρ and h are unknown, h can be expressed as a function of ρ and hence it follows that h = 2.864/ρ. If we take into account the tendency of melamine to stack at the typical stacking distances between aromatic rings (ca. 3.3 Å), applying this value gives a density of 0.87 g cm^{-3}. This density is reasonable for organic molecules of this type and supports the correct assignment of a 1:3 stoichiometry.
For other stoichiometry values, the deduced densities would be different from those expected for organic compounds. For example, for a 1:2 stoichiometric complex (molar mass of the complex 2597.2 g), the above equation would indicate that h = 1.984/ρ. This would give an estimated density of 0.60 g cm^{-3}, which is impossible for this type of organic compounds.

3. Preparation and Characterization of the Chiral Nanoporous Materials: M-dCouAzoC8-*r*-*CPL*-pol and M-dCouAzoC8-*l*-*CPL*-pol.

3.1. The preparation of the nanoporous material involves three steps as described in **Scheme S5.**

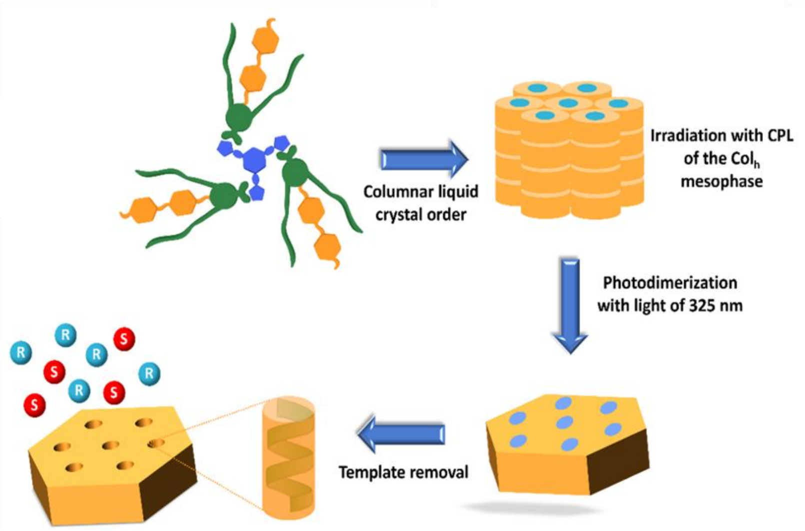

Scheme S5. *Step-by-step procedure for obtaining chiral nanoporous materials.*

3.2 Irradiation of M-dCouAzoC$_8$ in the columnar hexagonal phase with circular polarized phase to induce the helical organization in the column yielding the complexes M-dCouAzoC$_8$-*r-CPL* and M-dCouAzoC$_8$-*l-CPL*. 2 mg of the product **M-dCouAzoC$_8$-r-CPL** or **M-dCouAzoC$_8$-l-CPL** are placed and melted between two glasses that were previously spincoated (1500 rpm, 30s) with a solution of poly(vinyl alcohol) (1% in water) with spacers of 10 µm. The films were then heated above the isotropization temperature for 5 min and allowed to cool down room temperature at a rate of 0.01°C/min. The resulting films were irradiated for 1 minute with the corresponding CPL from the 488 nm line of an Ar^+ laser, power 20 mW cm^{-2}.

3.3 Preparation of the polymeric structures derived by M-dCouAzoC$_8$-*r*-CPL and M-dCouAzoC$_8$-*l*-CPL for photodimerization of the coumarin units: M-dCouAzoC$_8$-*r-CPL*-pol and M-dCouAzoC$_8$-*l-CPL*-pol.

The sandwich obtained in the previous treatment was irradiated with a 325 nm LED for 180 min and introduced into hot water until the **PVA** dissolved, obtaining the corresponding self-supporting polymeric materials (**M-dCouAzoC$_8$-r-CPL-pol** and **M-dCouAzoC$_8$-l-CPL-pol**).

3.4. Preparation of the nanoporous material by removal of the template molecules in the polymeric precursors: M-dCouAzoC$_8$-*r-CPL*-pol and **M-dCouAzoC$_8$-*l-CPL*-pol**

To remove the template molecules, the polymeric material was immersed in a solution of HCl/Ethanol 3M for 24h. Finally, the nanoporous material was washed with water and dried under vacuum, yielding **M-dCouAzoC$_8$-*r-CPL*-pol** and **M-dCouAzoC$_8$-*l-CPL*-pol**.

3.5. Complementary Figures

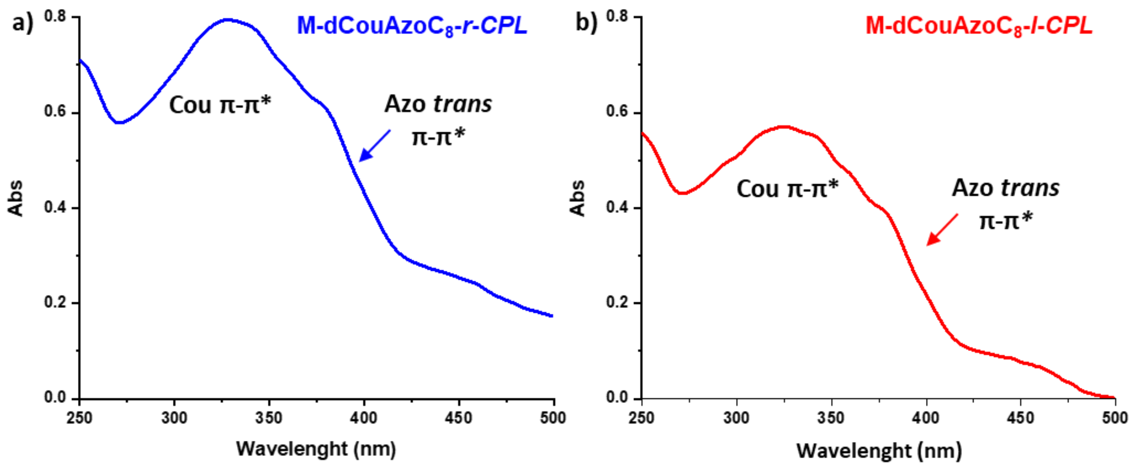

Figure S16. *UV spectrum of* **M-dCouAzoC$_8$** *after right* ***(r-*CPL*)*** ***(a)*** *and left* ***(l-*CPL*)*** ***(b)*** *circular polarized light irradiation.*

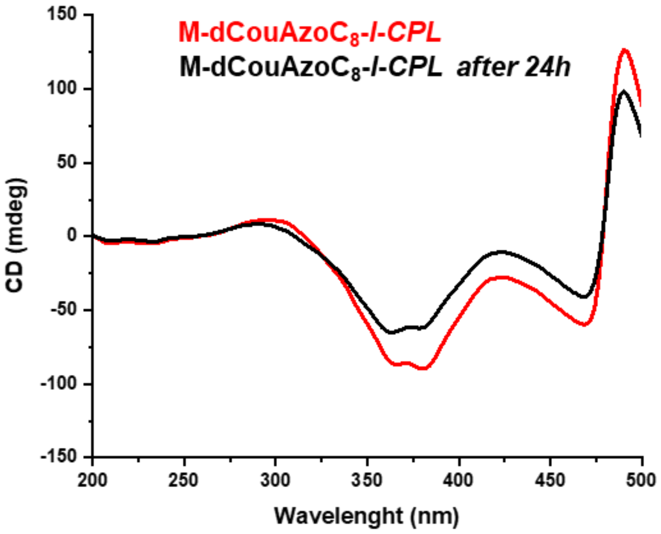

Figure S17. *CD spectra of* **M-dCouAzoC$_8$-*l*-CPL** *newly obtained (red line) and after 24h (black line).*

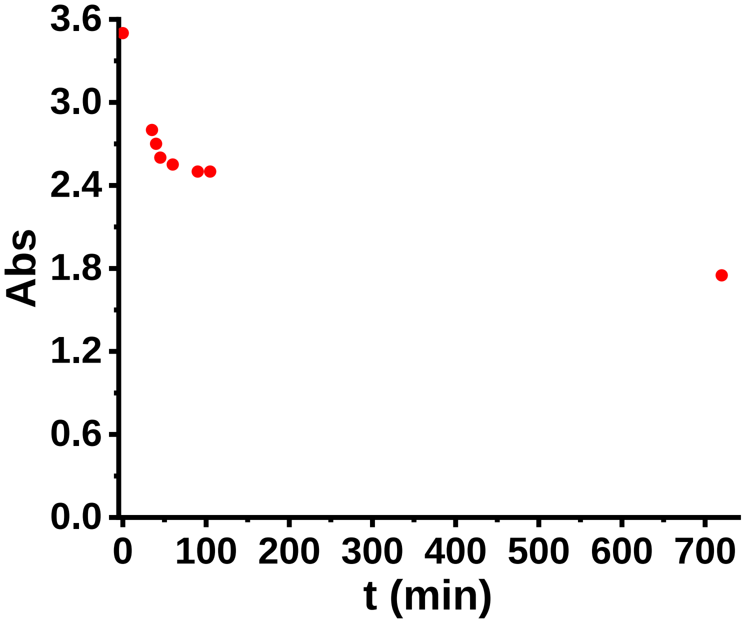

Figure S18. *Representation of the of absorption versus irradiation time for the photodimerization process of the compound* ***M-dCouAzoC$_8$-l-CPL***

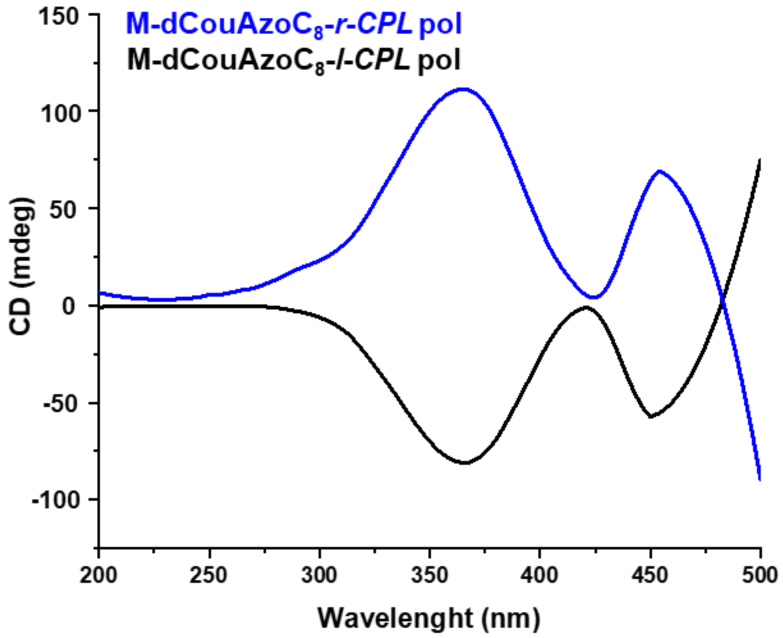

Figure S19. *CD spectrum of* **M-dCouAzoC$_8$-*r-CPL*** *and* ***l-CPL*** *after the photo-crosslinking process:* **M-dCouAzoC$_8$-*r-CPL*-pol** ***(blue line) and*** **M-dCouAzoC$_8$-*l-CPL*-pol** ***(black line)***.

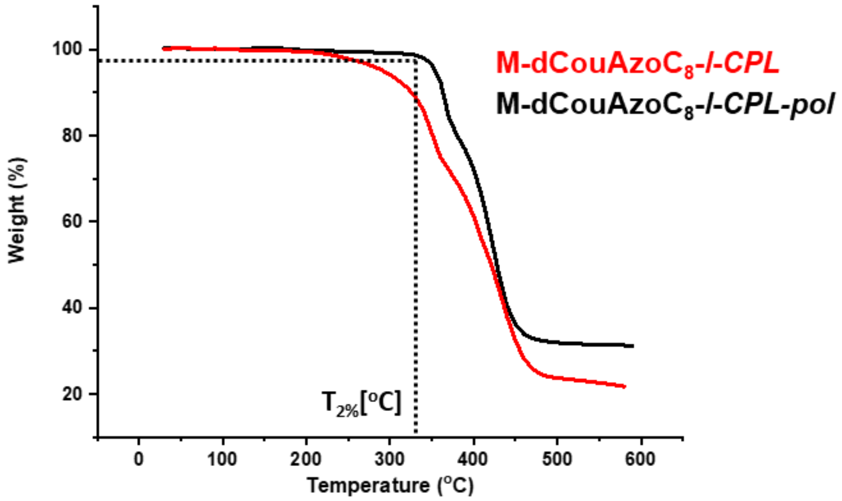

Figure S20. *TGA curves (10 °C min⁻¹)* of **M-dCouAzoC$_8$-*l-CPL*** *(red line) and* ***M-dCouAzoC$_8$-l-CPL-pol*** *(black line).*

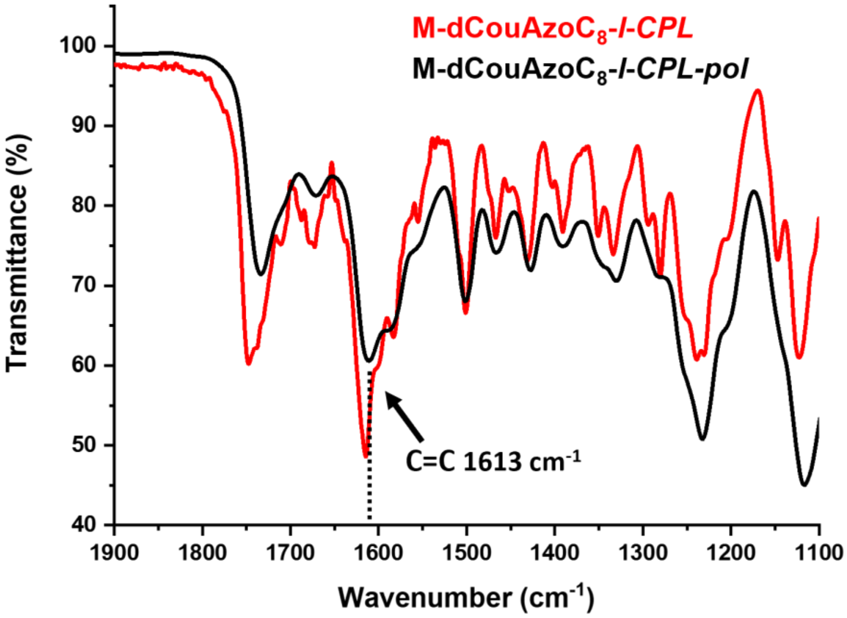

Figure S21. *FTIR comparison of* **M-dCouAzoC$_8$-*l-CPL*** *before (red) and after (black) photodimerization* **M-dCouAzoC$_8$-*l-CPL*-pol**.

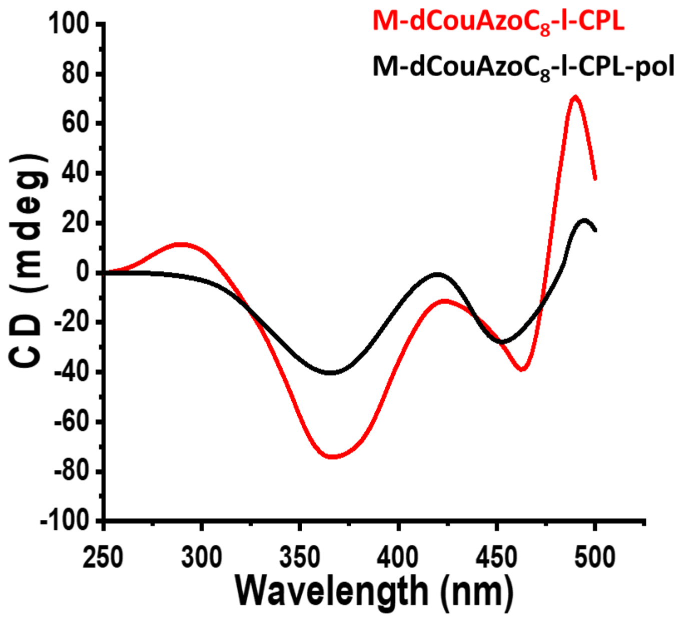

Figure S22. *Comparison between the CD spectrum of the* **M-dCouAzoC$_8$-*l*-*CPL*** *before (red) and after the photodimerization* **M-dCouAzoC$_8$-*l*-*CPL*** *(black).*

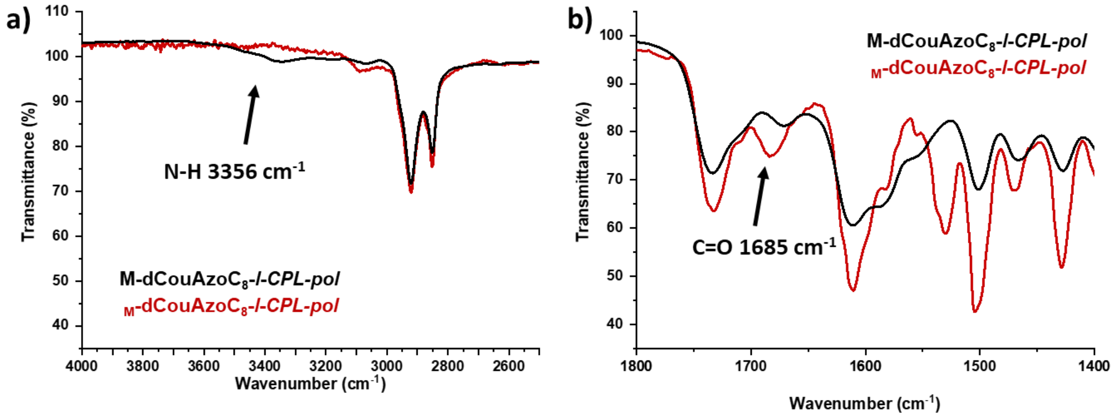

Figure S23. *FTIR comparison before of* **M-dCouAzoC$_8$-*l*-*CPL*-pol** *(black) and after* **$_{\text{M}}$-dCouAzoC$_8$-*l*-*CPL*-pol** *(dark red) acid treatment,* ***a)*** *amine region and* ***b)*** *carbonyl region.*

4. Adsorption capacity of the chiral nanoporous materials and kinetics of the process.

The adsorption capacity of the LC membranes was measured in relation of the milligrams of dye per gram of membrane as a function of time:

$$q_t = \frac{(C_0 - C_f)V}{m}$$

Where C_0 is the initial concentration (mg/L) of the dye, C_f is the final concentration (mg/L), V is the volume (L) of the solution and m is the mass of membrane (g). A value of 56.10 mg/g was obtained.

For a better understanding of the quantitative analysis, we tried to adjust these results to a first order kinetic adsorption whose equation is:

$$q_t = q_e(1 - e^{-kt})$$

Where q_e is the maximum concentration absorbed in the equilibrium, *t* is the time in that equilibrium and *k* is the kinetic constant. This model fits with precision for the adsorption capacity. As an example, in **Figure 6c** the curve, the equation and the R value are shown for the adsorption of nitroaniline derivative with **M-dCouAzoC$_8$.**
